# Cannabidiol in Foods and Food Supplements: Evaluation of Health Risks and Health Claims

**DOI:** 10.3390/nu17030489

**Published:** 2025-01-29

**Authors:** Barbara E. Engeli, Dirk W. Lachenmeier, Patrick Diel, Sabine Guth, Maria A. Villar Fernandez, Angelika Roth, Alfonso Lampen, Alexander T. Cartus, Wim Wätjen, Jan G. Hengstler, Angela Mally

**Affiliations:** 1 Federal Food Safety and Veterinary Office (FSVO), Division Knowledge Foundation, Section Risk Assessment, Schwarzenburgstr 155, 3003 Bern, Switzerland; barbara.engeli@blv.admin.ch; 2 Chemisches und Veterinäruntersuchungsamt (CVUA) Karlsruhe, Weißenburger Str. 3, 76187 Karlsruhe, Germany; lachenmeier@web.de; 3 Department of Molecular and Cellular Sports Medicine, Institute of Cardiovascular Research and Sports Medicine, German Sport University Cologne, Am Sportpark Müngersdorf 6, 50933 Cologne, Germany; diel@dshs-koeln.de; 4 Leibniz Research Centre for Working Environment and Human Factors (IfADo), Ardeystr. 67, 44139 Dortmund, Germany; guth@ifado.de (S.G.); villar-fernandez@ifado.de (M.A.V.F.); roth@ifado.de (A.R.); hengstler@ifado.de (J.G.H.); 5 Risk Assessment Strategies, Bundesinstitut für Risikobewertung (BfR), Max-Dohrn-Str. 8–10, 10589 Berlin, Germany; alfonso.lampen@bfr.bund.de; 6 Chemservice S.A., 13, Fausermillen, 6689 Mertert, Luxembourg; alexander.cartus@gmx.de; 7 Institut für Agrar-und Ernährungswissenschaften, Martin-Luther-Universität Halle-Wittenberg, Weinbergweg 22, 06120 Halle (Saale), Germany; wim.waetjen@landw.uni-halle.de; 8 Department of Toxicology, University of Würzburg, Versbacher Str. 9, 97078 Würzburg, Germany

**Keywords:** cannabidiol, CBD, food, food supplements, beneficial effects, health claims, health risk, hepatotoxicity, neurotoxicity

## Abstract

Background: Cannabidiol (CBD) is a cannabinoid present in the hemp plant (*Cannabis sativa* L.). Non-medicinal CBD oils with typically 5–40% CBD are advertised for various alleged positive health effects. While such foodstuffs containing cannabinoids are covered by the Novel Food Regulation in the European Union (EU), none of these products have yet been authorized. Nevertheless, they continue to be available on the European market. Methods: The Permanent Senate Commission on Food Safety (SKLM) of the German Research Foundation (DFG) reviewed the currently available data on adverse and potential beneficial effects of CBD in the dose range relevant for foods. Results: Increased liver enzyme activities were observed in healthy volunteers following administration of 4.3 mg CBD/kg bw/day and higher for 3–4 weeks. As lower doses were not tested, a no observed adverse effect level (NOAEL) could not be derived, and the dose of 4.3 mg/kg bw/day was identified as the lowest observed adverse effect level (LOAEL). Based on the CBD content and dose recommendations of CBD products on the market, the SKLM considered several exposure scenarios and concluded that the LOAEL for liver toxicity may be easily reached, e.g., via consumption of 30 drops of an oil containing 20% CBD, or even exceeded. A critical evaluation of the available data on potential beneficial health effects of CBD in the dose range at or below the LOAEL of 4.3 mg/kg bw/day revealed no scientific evidence that would substantiate health claims, e.g., in relation to physical performance, the cardiovascular, immune, and nervous system, anxiety, relaxation, stress, sleep, pain, or menstrual health. Conclusions: The SKLM concluded that consumption of CBD-containing foods/food supplements may not provide substantiated health benefits and may even pose a health risk to consumers.

## 1. Introduction

### 1.1. CBD as Lifestyle Product and Market Prevalence

Cannabidiol (CBD) is a cannabinoid present in hemp (*Cannabis sativa* L.). Most consumers use CBD in the form of non-medicinal over-the-counter products, so-called CBD oils, typically consisting of 5–40% CBD dissolved in an edible oil and advertised with a variety of health claims. CBD edibles such as beverages, cookies and sweets are also marketed. However, most of these products do not comply with the corresponding legal requirements and are thus sold in violation of food legislation. In the European Union (EU), foodstuffs including food supplements with cannabinoids are classified as novel foods [1] and require authorization by the European Commission based on a safety assessment by the European Food Safety Authority (EFSA). This applies to both extracted and chemically synthesized CBD. Thus far, all submissions of CBD as a novel food in the EU were incomplete; therefore, the authorization process is currently on hold until applicants submit the necessary data to cover the data gaps and safety concerns raised by EFSA [2].

Compared to the better-known psychotropic cannabinoid Δ^9^-tetrahydrocannabinol (Δ^9^-THC), CBD is not known to cause psychotropic effects, such as euphoria and altered perception. CBD is mostly obtained from low Δ^9^-THC-containing *Cannabis sativa* L. plant varieties but can also be synthesized chemically. CBD can be obtained from hemp using various extraction methods. This can lead to different cannabinoid profiles (CBD, Δ^9^-THC, and other cannabinoids) with different biological activity depending on the starting material (e.g., plant variety, plant part), solvent, and technique used [2]; therefore, interactions between structurally similar cannabinoids must be considered. In addition, Δ^9^-THC levels ranging from 0.1 to 0.3% are also frequently detected in CBD oils [3,4,5,6]. In a recent study, Δ^9^-THC was found in 78% of the samples (total samples *n* = 26) in a concentration range from 5 to 1576 mg/kg [4]. The authors noted that the Δ^9^-THC concentration in 50% of the products would exceed the acute reference dose (ARfD) of 1 μg/kg bw [7], taking into account the highest daily dose recommended by the manufacturers (20 drops) and a person with a body weight (bw) of 70 kg.

Additionally, deviations from declared CBD concentrations were reported by several studies with up to 226% of the declared value [4].

A recent study conducted in Germany reported that approximately 40% of the German population are aware of products containing CBD, while around 11% also use these products [8]. Additionally, this study indicated that consumers are insufficiently informed about products containing CBD, i.e., there is limited awareness of potential health risks and of the insufficient scientific data base for the claimed health benefits. According to German marketing materials on a CBD product website cited by Kraft et al. [9], the majority of German CBD consumers use CBD for the following purposes: pain relief (48%), relaxation (42%), general well-being (40%), sleep aid (31%), anti-inflammatory (23%), muscle relaxation after exercise (9%), and concentration improvement (6%). According to a survey of young adults in the US (*n* = 340), the top reasons for using CBD were stress relief (65%), relaxation (55%), and sleep improvement (42%) [10]. On English-language Twitter (X since July 2023), the top four claims for CBD are pain (32%), anxiety disorders (27%), sleep disorders (14%), and stress (10%) [11]. Pain, inflammation, and anxiety were the most common claims in surveys of CBD retailer websites in the USA [12,13]. A content analysis investigated how CBD products were presented on the social media platform Pinterest. The majority (91.6%) of pins described CBD positively, with many claiming a physical or mental benefit including pain, depression, anxiety, and inflammation relief [14]. Most pins (98.2%) did not mention possible side effects or recommend dosage. A survey in the UK found that users consume CBD to manage self-perceived anxiety, stress, sleep disorders, and other symptoms [15]. In a large review of 2165 CBD products from 70 websites in Canada, the most common claims found in product descriptions were the ability to treat or manage pain (*n* = 824), anxiety (*n* = 609), and inflammation (*n* = 545) [16]. It should be noted that there may be some overlap with therapeutic applications as a drug and that some of the claims identified may already fall within the boundaries of drug indications. Nevertheless, any health claims on foodstuffs require scientific substantiation in Europe, which has neither been requested nor granted so far for CBD.

### 1.2. CBD as a Medicinal Drug

At present, the only authorized oral medicinal CBD mono-preparation on the EU market is Epidyolex^®^ (Amersfoort, The Netherlands) (or Epidiolex^®^ outside the EU) [2]. The active ingredient is a CBD extract (≥98% purity) derived from *Cannabis sativa* L., dissolved in sesame oil. It has been developed as an orphan drug and was positively evaluated by the US Food and Drug Administration (FDA) [17] and the European Medicines Agency (EMA) [18] and approved by the European Commission [19] for the treatment of severe childhood epilepsy (e.g., Lennox–Gastaut syndrome, Dravet syndrome, tuberous sclerosis complex). The therapeutic starting dose of Epidyolex^®^ is 5 mg CBD/kg bw/day administered orally in two daily doses of 2 × 2.5 mg CBD/kg bw/day (e.g., 300 or 350 mg for a person weighing 60 or 70 kg, respectively), which is subsequently scaled up to up to 25 mg CBD/kg bw/day in two daily doses of 2 × 12.5 mg CBD/kg bw/day (e.g., 1500 or 1750 mg for a person weighing 60 or 70 kg, respectively). Regarding medical applications, undesirable effects may be accepted if the benefits outweigh the potential negative effects. By contrast, adverse effects of foodstuffs are not acceptable.

### 1.3. Novel Food Status and Regulations

CBD is considered a novel food in the EU. According to EU Regulation No. 2015/2283, a novel food is defined as a food that has not been consumed to a significant degree by humans in the EU before May 1997. Isolated CBD or CBD contained in hemp extracts were not consumed prior to this date and are therefore considered as novel food [1,20]. This means that CBD products must undergo a safety assessment by EFSA and be approved by the European Commission before they can be sold in the EU. In addition, CBD products must also comply with EU food safety and labelling regulations. EFSA has reviewed novel food applications for CBD and various *Cannabis*-derived products. However, these reviews have been put on hold and are in a state of “clock stop” due to insufficient data submitted by industry to confirm the safety of the products [2,21]. In addition, available data indicate safety concerns [2].

Despite the lack of novel food approval, CBD products are on the market in the EU, because companies intentionally sell CBD products even without mandatory authorization. However, it is difficult for the regulatory authorities to enforce the regulation applying to foodstuffs [3]. The situation is similar in the US, where the FDA has not yet established a clear regulatory framework for CBD, and products containing CBD are being sold without FDA approval and despite safety concerns raised by the FDA [22,23]. The UK Food Standards Agency (FSA) recently derived a provisional acceptable daily intake (ADI) for CBD (>98% purity, from hemp extracts or synthetic) as novel food of 10 mg CBD/day (corresponding to 0.14 mg/kg bw/day for a 70 kg person) [24]. In Switzerland, the situation is the same as in the EU with no approved novel food so far. To enable control authorities in their enforcement of the food law, a limit of 12 mg CBD/person (70 kg bw) per day was established in such products based on hepatoxic effects in humans [25].

### 1.4. Concept/Aim

The Permanent Senate Commission on Food Safety (SKLM) of the German Research Foundation (DFG) discussed the current data with respect to potential health risks and benefits of pure CBD (approx. 98%) used in food or food supplements. Various human studies with healthy subjects have demonstrated that oral ingestion of CBD for 4 weeks at doses ≥ 4.3–5 mg/kg bw per day (corresponding to 150 mg CBD twice per day for a 70 or 60 kg adult, respectively) produces adverse effects in the liver [2,25,26,27]. Furthermore, in controlled clinical trials for the approval of Epidyolex^®^, oral CBD doses above 5 mg/kg bw per day resulted in adverse effects, e.g., on the central nervous system (CNS) (somnolence, sedation), the gastrointestinal tract (e.g., decreased appetite and diarrhoea) and infections (e.g., pneumonia) [2,17,18]. Based on the available human data, 300 mg CBD/day (4.3 or 5 mg CBD/kg bw/day for a 70 or 60 kg adult, respectively), can be considered the “lowest observed adverse effect level” (LOAEL) in humans, while a “no observed adverse effect level” (NOAEL) cannot be derived. As adverse effects of foodstuffs are not acceptable, it follows that intake of CBD via food/food supplements must be well below the current LOAEL. There is also a need to understand if positive effects claimed for CBD-containing foods/food supplements in this dose range are supported by scientific evidence. The aim of this narrative review is therefore to assess if CBD at doses below the LOAEL of 4.3–5 mg/kg bw per day (<300 mg/day) causes scientifically reproducible positive health effects in humans after oral intake of CBD-containing foods/food supplements, to analyze if and by which degree the LOAEL of 4.3–5 mg/kg bw/day is currently exceeded by consumers, as well as to weigh potential benefits against the adverse effects in a risk–benefit analysis.

## 2. Methods

A narrative review was conducted to provide an up-to-date scientific assessment of the potential health risks and benefits of CBD in foods and food supplements following the EFSA guidance on risk–benefit assessment of foods [28]. This guidance recommends using the available assessments of health authorities and the available systematic reviews. The starting point for the characterization of adverse effects of CBD was the current statement on the safety of CBD as a novel food by EFSA in 2022 [2]. The human and animal studies already cited in the EFSA assessment are only briefly summarized in the individual risk Section 5.1, Section 5.2, Section 5.3, Section 5.4, Section 5.5, Section 5.6, Section 5.7 and Section 5.8. Additional studies published by February 2024 were also included in the risk sections.

The focus of the benefit analysis was on the effects expected at doses achieved via consumption of foods/food supplements, while studies on pharmacological activity at doses higher than 4.3–5 mg/kg bw/day (300 mg/person/day), the oral therapeutic starting dose of Epidyolex^®^, were not considered. A dose of 4.3–5 mg/kg bw/day was also the lowest dose tested in the Epidyolex^®^ dossier and in several other human studies in which this dose produced adverse effects on the liver and CNS. A literature search was conducted in PubMed (Medline) and Web of Science up to February 2024, covering various potential beneficial effects and health claims, and further literature was searched via citation tracking. Health claims were selected based on the most frequent mentions on industry websites and health portals, e.g., [29]. The authors reviewed the results of the literature search using the following inclusion criteria: The focus was on human studies in which pure CBD (approx. 98%) was administered orally to healthy individuals at doses at or below 300 mg/day (equivalent to 4.3–5 mg/kg bw/day for a person weighing 70 or 60 kg, respectively). Studies in a dose range above 300 mg CBD/day were also briefly summarized, when available and relevant, to provide an overview of whether beneficial effects were observed in the higher therapeutic dose range. Studies in patients with a focus on medical use were generally excluded, but in some cases were described to complement the overall assessment, for example, when secondary effects were investigated. In such cases, the patients’ health condition and whether they took concomitant drugs was reported. Studies in which *Cannabis*, a combination of CBD and Δ^9^-THC, or unspecified hemp extracts were administered were not included into the assessment. The results of human studies with healthy volunteers and oral treatment with pure CBD in a dose range below 300 mg CBD/day were summarized in separate tables for each of the health claims investigated. Results from animal studies, preferably using oral administration, were also included, but other routes of exposure (intraperitoneal injection (i.p.), intravenous injection (i.v.), dermal, inhalation) were also taken into account if considered relevant.

The risk–benefit assessment was carried out on the basis of the EFSA guidance on risk–benefit assessment of foods [28], which recommends a tiered approach: Tier 1 involves an initial risk–benefit assessment to clarify whether the health risks clearly outweigh the health benefits (risks >> benefits), i.e., whether the risks already occur at low exposures, while a benefit is only recognizable at high exposures, or the benefits clearly outweigh the risks (risks << benefits), i.e., whether the benefits can already be expected at low exposures, while the risks only occur at high exposures [28]. Tier 2 refines the assessment using non-effect size-based measures of risks and benefits in order to provide semi-quantitative or quantitative estimates at relevant exposure [28]. Tier 3 further refines the assessment using effect size-based measures of risks and benefits and compares them using a composite metric such as DALYs (Disability-Adjusted Life Year) or QALYs (Quality-Adjusted Life Year) to convey the results of the risk–benefit evaluation as a single net health outcome value [28]. In the present study, only the initial assessment (Tier 1) was carried out in accordance with the EFSA guideline, which stipulates that no further action (i.e., no refined assessment) is required if the initial assessment leads to a definitive conclusion [28]. The risks and benefits were examined separately, but no specific health metrics were used. Instead, exposure was compared to established health-based guidance values (HBGVs), such as the ADI or tolerable daily intake (TDI), for risk and minimum dose levels associated with a beneficial health effect. As this analysis revealed that the risks clearly outweigh the benefits when considering the exposure scenarios relevant for foods/food supplements (see Section 7 and Section 10), no refined risk–benefit assessment was performed.

## 3. Molecular Targets of CBD and Putative (Pharmacological) Mode of Action

**Figure 1:**
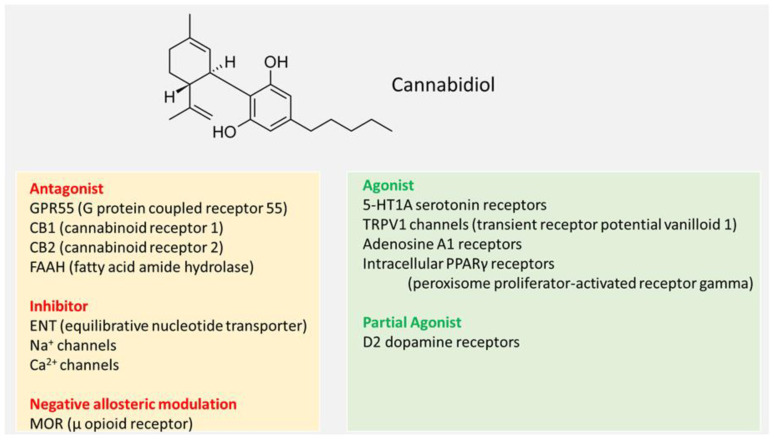
Examples of different molecular effects of CBD (modified from [31]).

Molecular targets of CBD were described in detail by EFSA [2] and in several reviews [30,31,32,33,34]. CBD is assumed to interact with various molecular targets (Figure 1) that are widely distributed throughout the body, e.g., the gut, brain, muscle, heart, adipose tissue, bone, and the endocrine system [2]. Thus, potential effects of CBD are thought to be mediated through multiple molecular modes of action (MoAs). The compound modulates receptor targets, e.g., GPR55; enzymes, e.g., cytochrome P450 (CYP450); ion channels, e.g., transient receptor potential (TRP) channels; and transporters, e.g., anandamide transporters. Moreover, CBD modifies oxidative stress and inflammation.

CBD is an antagonist of cannabinoid receptors CB1 and CB2 [2]. In addition, CBD may have an indirect effect on cannabinoid receptors by inhibiting the activity of fatty acid amide hydrolase (FAAH), a major enzyme involved in the degradation of endogenous cannabinoids, e.g., anandamide, the main endogenous CB1 receptor agonist [2,31]. Furthermore, CBD showed antagonistic effects at GPR55, a G-protein-coupled receptor proposed as a third cannabinoid receptor [2]. This can lead to overexpression of endo-cannabinoids and interleukin 10 [2]. Agonistic effects at the serotonin receptor 5-HT1A and transient receptor potential vanilloid 1 (TRPV1) channels may contribute to anxiolytic and analgesic effects [31]. It has also been shown that CBD acts as a partial agonist of D2 dopamine receptors and could thus have an antipsychotic effect [2,31]. CBD has also been reported to act as a full agonist of adenosine A1 receptors, which could have a positive effect on cardiac arrythmias and ischemia/reperfusion lesions in the myocardium [31]. CBD has been proposed to act as a competitive inhibitor of the adenosine transporter on EOC-20 microglial cells, thereby increasing the endogenous adenosine content [2]. Moreover, CBD has been shown to behave as a negative allosteric modulator of l- and d-opioid receptors and as a positive allosteric modulator of gamma-aminobutyric acid type A (GABA-A) receptors, which could contribute to its anxiolytic, anti-seizure, and analgesic effects [2,31]. CBD exerted agonistic activities on peroxisome proliferator-activated receptor gamma (PPAR-gamma) receptors, which act on lipid storage and glucose metabolism [2,31]. In addition, CBD inhibits sodium and calcium channels and thus shows a modulating effect on the membrane potential, making CBD a possible therapeutic agent for the treatment of epilepsy [31]. CBD is reported to be an effective regulator of intracellular redox balance, exhibiting both antioxidant and pro-oxidative effects in cellular systems. The antioxidative effects are due to modulation of nuclear factor erythroid-2-related factor 2 (Nrf2)-signaling, whereas the pro-oxidative effects are thought to be mediated, e.g., by mitochondrial dysfunction and stimulation of cell death. CBD affects proteins involved in the Nrf2 (Keap1, MAPKs, GSK3β, SIRT1) as well as the NF-κB (nuclear factor kappa-light-chain-enhancer of activated B cells) pathway. CBD was shown to decrease the level of several pro-inflammatory cytokines (tumor necrosis factor α (TNF-α), interleukin 1β (IL-1β)) by inhibiting the NF-κB pathway in lipopolysaccharide (LPS)-treated cells. At high concentrations, CBD was reported to contribute to anti-inflammatory effects via inhibition of the transcription of pro-inflammatory genes such as inducible nitric oxide synthase (iNOS) and cyclooxygenase-2 (COX-2). CBD was reported to inhibit CYP450 enzymes, thereby affecting metabolic processes, and also to inhibit enzymes such as acetylcholinesterase (AChE), thereby affecting neurotransmitter concentrations [35].

The oxidation of CBD can lead to the formation of various metabolites, with CBD-hydroxyquinone (CBD-HQ) being one of the primary oxidation products of particular interest due to its potential biological effects. CBD-HQ can be formed during improper storage conditions but also through microsomal metabolism in hepatocytes [36]. While CBD itself demonstrated cytotoxicity in colon cancer cells with an IC_50_ (half-maximal inhibitory concentration) of 4.13 µg/mL, CBD-HQ exhibited comparable but slightly lower potency with an IC_50_ of 8.00 µg/mL [36]. Interestingly, CBD-HQ showed stronger cytotoxic effects in 3D cell culture models compared to CBD, suggesting potentially greater efficacy in vivo [36]. To our knowledge, it is unclear whether the conversion of CBD to CBD-HQ in biological systems is associated with increased reactive oxygen species (ROS) production [37], which could contribute to hepatotoxicity or whether it has antioxidant properties and reduces intracellular ROS levels, as shown by Beben et al. [36] in colon cancer cells. Furthermore, disruptions in cell cycle, apoptosis, and endoplasmic reticulum (ER) stress might be involved in the hepatic cytotoxicity induced by CBD [38].

Overall, CBD can interfere with numerous receptors and signaling pathways both in vitro (in a concentration range of 0.01–100 µM) and in vivo, triggering a myriad of biological effects [2]. In its scientific opinion, EFSA highlighted the necessity of considering these receptor interactions when assessing the safety of CBD, also considering the differences in study design (including model system, assays, and concentrations), as well as the interspecies variations in receptor distribution [2].

## 4. Absorption, Distribution, Metabolism, and Excretion (ADME)

The oral pharmacokinetics of CBD in humans have been investigated in clinical trials on the drug Epidyolex^®^ [39]. Information from these and further studies recently reviewed by EFSA (2022) are briefly summarized here [2].

Oral bioavailability of CBD is low and variable (average 6%), owing to poor solubility of highly lipophilic CBD and pre-systemic biotransformation. Oral bioavailability of CBD appears to depend to a significant extent on the matrix used to deliver CBD. Concomitant consumption of dietary fats has been shown to significantly increase the solubility and absorption of CBD, resulting in ~5-fold increase in maximum plasma concentrations (c_max_) and a ~4-fold increase in the area under the curve (AUC) compared to the fasted state [39]. Concomitant consumption of a low caloric diet, milk, and alcohol also increased the oral bioavailability and c_max_.

In oral pharmacokinetic studies on Epidyolex^®^, the median time of CBD to reach the c_max_ (t_max_) was reported to be 2.5–5 h [39]. A similar range (t_max_~2–5 h) was reported following oral administration of a single dose of CBD embedded in gelatine matrix beadlets, with significant interindividual differences [40].

CBD binds to serum albumin (94–99%) [41], is rapidly distributed, and accumulates in adipose tissues. Animal experiments suggest that it can cross the blood–brain barrier [41,42]. CBD undergoes extensive pre-systemic biotransformation in the gut and the liver. CYP450-mediated oxidation, primarily via CYP2C19 and CYP3A4, gives rise to 7-hydroxy-cannabidiol (7-OH-CBD) and 7-carboxy-cannabidiol (7-COOH-CBD) as major metabolites [39,41]. Glucuronidation of CBD at the phenolic group via UDP-glucuronosyltransferase (UGT1A7, UGT1A9, and UGT2B7 enzymes) is a further route of CBD biotransformation [41]. Following CBD administration twice a day for 7 days, the mean elimination half-life was reported to range between 56 and 61 h. Elimination of CBD was reported to occur primarily via feces (84%) and to a lesser extent via urine [39].

## 5. Characterization of Adverse Health Effects of CBD

In 2022, EFSA identified the risks associated with the use of CBD as a food supplement and/or food ingredient and summarized the uncertainties and data gaps that remain to be solved before concluding the safety assessment of CBD as a novel food [2]. The following sections briefly summarize the conclusions drawn by EFSA as well as data published since then. Table 1 presents a brief overview of adverse health effects of CBD. To ensure comparability across studies, doses are expressed both in mg per person per day and in mg per kg bw per day, assuming a bw of 70 kg if not otherwise stated in the publication.

### 5.1. Liver

#### 5.1.1. Animal Data

As summarized by EFSA (2022), repeated oral dose toxicity studies conducted in mice, rats, dogs, and rhesus monkeys applying CBD in the form of CBD extracts of varying purity for different study durations (from 10 days up to 39 weeks) consistently revealed evidence for adverse effects of CBD in the liver [2]. Effects of CBD on the liver included increased liver weight (both absolute and relative weights), hypertrophy of liver cells, increased serum liver enzyme activities (alanine aminotransferase (ALT), aspartate transaminase (AST), alkaline phosphatase (ALP), gamma-glutamyl transferase (GGT)) or increased bilirubin, although the pattern of effects reported varied between studies [2]. It should be taken in mind that the use of hemp extracts in CBD products (so-called full-spectrum or broad-spectrum CBD) may result in interactions between the cannabinoids, which could influence their adverse effects in a product-specific manner.

EFSA did not identify a NOAEL for endpoints related to liver effects. A LOAEL of 10 mg/kg bw/day (lowest dose tested) was reported in a 39-week study in dogs with highly purified CBD [2].

Recently performed OECD/GLP-compliant rat studies with oral (gavage) administration of a hemp-derived CBD-isolate (>99% purity, in olive oil) for 14 days (0, 30, 70, or 150 mg CBD/kg bw/day), 90 days with 28 days recovery (0, 50, 80, 120, or 140 mg CBD/kg bw/day), as well as a reproduction/developmental screening toxicity study (0, 30, 100, or 300 mg/kg bw/day) by the US Canopy Growth Corporation are now available [43,44]. These studies confirm the liver effects in both sexes, with increased absolute and relative liver weights and centrilobular hepatocellular hypertrophy observed at the end of the study (e.g., 90-day study: elevated absolute and relative liver weight from 80 mg/kg bw/day in males and from 120 mg/kg bw/day in females; hepatocellular hypertrophy in both sexes from 80 mg/kg bw/day, which recovered in the 28-day recovery group). Liver enzyme activities at the end of the study were either not elevated in the 14- and 90-day studies or not analyzed in the reproduction/developmental toxicity study. Similar results were also obtained in another recent 90-day OECD/GLP rat study with oral (gavage) administration of a hemp-derived CBD isolate (>95% purity, ethanolic extract of whole *C. sativa* L. plant, in medium chain triglycerides) administered at 0, 30, 115, 230, and 460 mg/kg bw/day [45]. At doses ≥ 115 mg/kg bw/day, relative liver weight was elevated in both sexes. These effects resolved in male rats after the 35-day recovery period (examined at 460 mg/kg bw/day). However, relative liver weight was still elevated in the corresponding female recovery group. Centrilobular hepatocellular hypertrophy occurred in males at 115 mg/kg bw/day and in females at 230 mg/kg bw/day. The only statistically significant change in liver enzyme activities at the end of the treatment was elevated ALT in females at 460 mg/kg bw/day.

The authors of both studies set the highest tested dose as the NOAEL and argued that the observed effects on the liver were reversible and therefore adaptive and non-adverse. However, it is controversial whether these effects on the liver can be regarded as adaptive and non-adverse, so that the validity of the suggested NOAEL is questionable. The changes may initially be reversible, but may result in chronic inflammation, fibrosis, and subsequently permanent liver damage [69]. It is noteworthy that reversible effects on the liver were also observed in the human studies with Epidyolex^®^, as well as in other studies [18], and, as a result, standard monitoring of liver cell function during therapy was proposed in the EMA assessment report [18]. It should also be borne in mind that such effects may be tolerated for medicinal products that are only taken for a limited period of time, but may not be acceptable for foods/food supplements where lifelong consumption can be assumed and where the safety of a lifelong, daily intake without monitoring of liver parameters must be guaranteed [21]. Furthermore, the WHO recognized that the relevance of the reversibility of a toxic effect depends on the pattern of human exposure [70]. Thus, if exposure to a particular substance in the diet is expected to occur on a regular/daily basis, the potential risk would remain, regardless of the reversibility of the effects [70].

#### 5.1.2. Human Data in Healthy Volunteers

##### Therapeutic Doses (1500 mg CBD/Person per Day)

In a study aimed to assess the safety of Epidyolex^®^ among healthy volunteers, 16 male and female adults were administered the therapeutic dose of Epidyolex^®^ of 1500 mg CBD/person/day (twice daily 750 mg, corresponding to ~20 mg CBD/kg bw/day) for 27 days, including a phase-in-period of 11 days [46,47]. The serum levels of AST, ALT, ALP, GGT, and bilirubin were monitored during the study. In 7 out of 16 subjects (~44%), ALT exceeded the upper limit of normal (ULN) levels. In five of the subjects (~31%) ALT was even >5× ULN along with elevated levels of AST, ALP and GGT, while bilirubin remained unchanged. Changes occurred within 2–4 weeks after CBD administration and were reversible after discontinuation of CBD, except for one case of elevated GGT (GGT 2.4 × ULN at follow-up visit, ~14 days after last CBD-dose) [47]. Among the six participants who discontinued the study due to elevated liver enzyme activities, some showed symptoms consistent with hypersensitivity reactions or hepatitis. No correlation between transaminase increases and baseline characteristics, CYP2C19 genotype, or CBD plasma levels was found. Watkins et al. [46] pointed out that the ULNs for ALT (68 IU/L for both men and women) were roughly double than those reported in an international consensus document (25 IU/L for women, 33 IU/L for men [71]). Considering these consensus ULNs, 69% of the subjects had elevated ALT concentrations (as opposed to 44%, including one subject with ALT > 20× ULN).

In a randomized study on abrupt CBD withdrawal, 30 healthy male and female adult volunteers received 2 × 750 mg highly pure CBD (Epidyolex^®^)/person/day (corresponding to ~20 mg CBD/kg bw/day) orally for 28 days, followed by 2 × 750 mg highly pure CBD (Epidyolex^®^)/person/day orally for 14 days (arm 1, *n* = 9) or placebo for 14 days (arm 2, *n* = 12) [48]. Two participants (6.7%) were withdrawn for potential drug-induced liver injury (DILI) with increased transaminase activities and associated signs and symptoms during the first 28 days of treatment. In addition to these 2 cases, 12 volunteers also had increased transaminase activities, albeit below 3× ULN. There was no group not receiving any CBD during the first 28 days.

##### Therapeutic Starting Dose or Lower (≤300 mg CBD/Person per Day)

In a randomized controlled trial in healthcare workers being treated for emotional exhaustion and burnout throughout the COVID-19 pandemic, 59 healthy adults (males and females) received 300 mg CBD per person (99.6% purity, 150 mg twice daily; corresponding to ~4.3 mg/kg bw/day) for 4 weeks additionally to standard care versus 59 receiving exclusively standard care (control) [72]. Standard care did not comprise any medication potentially interacting with CBD, according to EFSA 2022 [2]. ALT, AST, GGT, ALP, and bilirubin in serum were assessed. Four participants (6.8%) had increased liver enzymes (>3-fold above the ULN), resulting in the discontinuation of the treatment for one subject (not further specified which liver enzyme(s); however, according to the supplementary data of the publication [72], mean ALT and AST of the CBD group were elevated at the end of the study compared to baseline). The authors reported that total bilirubin levels did not rise more than twofold in any of the participants. No increases in liver enzyme activities were seen in the control group.

The Epidyolex^®^ submission to the US FDA briefly mentions a phase I trial in which 5 out of 12 healthy subjects receiving 5 mg CBD/kg bw/day for 3 weeks experienced ALT elevations > ULN [73]. In the context of the authorization process of Epidyolex^®^, increased liver enzyme activities were reported at the same dose range in patients with epilepsy (Lennox–Gastaut or Dravet syndrome) concomitantly using antiepileptic medication. The main safety issue was hepatotoxicity, which in some instances was severe and serious, resulting in hospitalization [18].

Overall, increased liver enzyme activities (mainly ALT and AST) were observed in healthy human volunteers orally administered highly pure CBD at 20 mg/kg bw/day and in one study at 4.3 mg/kg bw/day for 3–4 weeks. As no lower doses were tested, there is no NOAEL available, and 4.3 mg/kg bw/day is considered the LOAEL for liver toxicity in healthy humans.

### 5.2. Gastrointestinal Tract

Adverse effects of CBD on the gastrointestinal tract have recently been reviewed and summarized [2]. A series of studies in healthy adults and patients suffering from epilepsy or seizures receiving either highly purified plant-derived CBD or a non-natural isomer (+)CBD consistently reported an increase in the occurrence of diarrhea as a frequent adverse effect of CBD. These included five randomized control trials in patients with epilepsy or seizures taking CBD at an oral dose of 10–50 mg/kg bw/day (taken as two separate doses) for 2–16 weeks. Results suggest a dose-dependent increase in the occurrence of diarrhea of up to 57% at the highest dose [49,50,51,52,53]. A similar occurrence of diarrhea (50%) was reported in an open-label clinical trial [46], in which 16 healthy adults received CBD in the form of the drug Epidyolex^®^ for 27 days at escalating doses up to 1500 mg (corresponding to ~20 mg/kg bw/day). There is also evidence for a dose-dependent effect from randomized control trials in presumably healthy adults. In one study, 7.1% and 23% of subjects with heroin use disorder taking 400 or 800 mg/day CBD (corresponding to approximately 5 and 10 mg/kg bw/day; mean bw 80 kg) for 3 days suffered from diarrhea, whereas the occurrence in the corresponding placebo group was 0% [74]. Similarly, in a randomized control trial by Taylor et al. [54] in which presumably healthy adults were given placebo or CBD at 750 and 1500 mg/day for 7 days (~10.7 and 21.4 mg/kg bw/day; taken as two separate doses on days 1–6 and a single dose on day 7), the occurrence of diarrhea increased in a dose-dependent manner (44% and 88.9% in the 750 and 1500 mg/day dose group, respectively), compared to 0% in the placebo group. Results from a further arm of the study in which subjects received a single dose of 1500 mg CBD (~21.4 mg/kg bw/day) suggest that the occurrence of diarrhea may depend on the fasting/non-fasting state [54]. In the fasted state, diarrhea occurred in 25% of subjects, whereas none of the subjects consuming CBD in the fed state suffered from diarrhea. Perkins et al. (2020) also reported diarrhea as the most frequently reported adverse effect, besides headache, following oral intake of CBD at 5–10 mg/kg bw [55]. In contrast to these studies, no increase in the occurrence of diarrhea was reported in two randomized control trials at 200–800 mg CBD/day (~3–11 mg/kg bw/day) [56,72], although Arout et al. (2022) reported a treatment-related increase in upset stomach (e.g., flatulence and abdominal cramps) [56].

While these studies provide evidence for diarrhea as a frequent adverse effect of CBD, EFSA noted a lack of studies investigating gastrointestinal effects of acute and long-term exposure to CBD in healthy subjects [2]. The mechanistic basis for the gastrointestinal symptoms associated with CBD intake is unknown.

### 5.3. Neurological, Psychiatric, and Psychological Effects

There are few studies with healthy volunteers that assessed adverse effects of CBD on the nervous system.

In a study by Taylor et al. with 30 healthy male and female adult volunteers receiving 2 × 750 mg CBD (Epidyolex^®^)/person/day (~20 mg/kg bw/day) orally for 28 days, the nervous system disorders reported were: headache (15/30, 50%), somnolence (7/30, 23.3%), dizziness (7/30, 23.3%), disturbance in attention (2/30, 6.7%), postural dizziness (2/30, 6.7%), nightmares (2/30, 6.7%), and insomnia (2/30, 6.7%) [48]. A control group that did not receive CBD during the 28 days was not included in this study, as its aim was to assess withdrawal symptoms induced by abrupt cessation of CBD.

In a previous study by Taylor et al., fasted healthy male and female subjects received CBD (Epidyolex^®^) orally for 7 days, with two doses of 750 or 1500 mg CBD per day (~20 mg/kg bw/day) up to day 6, and a single dose on day 7 (*n* = 9 subjects per dose group) or placebo (*n* = 6 subjects) [54]. Reported adverse events concerning the nervous system or psychiatric disorders included the following: headache (44.4% in each CBD group vs. 0% placebo), somnolence (44% of participants administered 1500 mg CBD twice daily vs. 33.3% placebo), dizziness (33.3% of participants administered 1500 mg CBD twice daily vs. 0% placebo), postural dizziness (22.2% of participants administered 1500 mg CBD twice daily vs 0% placebo), and insomnia (22.2% of subjects receiving 1500 mg CBD twice daily vs 0% placebo).

In a double-blind study, 10 healthy male participants received a single oral dose of 400 mg (~6 mg/kg bw/day) 99.9% pure CBD or placebo (as capsules) in a crossover design 1 week apart [57]. CBD administration was linked to a statistically significant increase in mental sedation and reduction of subjective anxiety, as assessed by visual analogue mood scale prior to an anxiety-evoking situation (cannula-insertion and single-photon emission computed tomography (SPECT)-scanning, 60 and 75 min after CBD intake) compared to placebo within the same subjects.

With regards to studies in patients, controlled clinical trials with oral doses of 5, 10, or 20 mg highly pure CBD (Epidyolex^®^)/kg bw/day were conducted in connection with the authorization of Epidyolex^®^ as a supplementary antiepileptic drug together with one or more approved antiepileptic drugs for the treatment of seizures in patients with Dravet or Lennox–Gastaut syndrome. Sedation, somnolence, lethargy, ataxia, abnormal motor coordination, irritability, aggression, anger, insomnia, and sleep disorders were reported at higher incidences in individuals who had received Epidyolex^®^ compared to placebo. Some of these adverse events (abnormal coordination, ataxia, lethargy, sedation, somnolence) were reported already at a dose of 5 mg CBD/kg bw/day. Besides increased liver transaminase activities, somnolence and sedation were the main reasons for study discontinuation (summarized by [2] with reference to FDA 2018 and [18]).

### 5.4. Endocrine System

Endogenous cannabinoids can influence the regulation of energy homeostasis and the endocrine system, e.g., the activity of the thyroid and pituitary glands, the pancreas and the adrenal cortex [75,76]. CB1 and CB2 activation, also by exogenous cannabinoids or consumption of cannabis, affects the secretion of prolactin [77], inhibits the secretion of growth hormone [78], luteinizing hormone (LH) [79], the secretion of estradiol and progesterone [80], testosterone in men [81] and vasopressin [82]. Further targets are the regulation of glucose homeostasis and adipogenesis [83]. Based on these data, there is clear evidence that cannabinoids can affect the endocrine system in many ways. This is being discussed as an opportunity for the development of pharmacological concepts for treatment of endocrine disorders. Moreover, these data suggest that cannabinoids can also be defined as endocrines disruptors. Regarding the specific effects of CBD on the endocrine system, there is a lack of data, which was also highlighted by EFSA [2].

#### 5.4.1. Hypothalamic–Pituitary–Gonadal Axis

According to EFSA [2] there is evidence from oral in vivo toxicity studies in sexually mature murine and simian models that CBD can influence gonadotropin (including luteinizing hormone and follicle-stimulating hormone) and sex hormone (including, testosterone, estradiol, and progesterone) levels, in males and females [58,60,61]. However, the mode of action of CBD on the endocrine system and its relevance to humans remain unclear [2]. The possible mechanisms proposed by EFSA include a direct effect of CBD on the endocannabinoid pathway(s) in the testes and liver and/or an interaction with certain CYP450 isoenzymes responsible for the metabolization of testosterone in the liver and for steroidogenesis [2].

There are no data on hormonal levels from the safety assessment of Epidyolex^®^ [2,18]. However, EMA risk assessors concluded that clinical and pharmacovigilance activities are necessary to enable monitoring for possible endocrine disruption [2,18].

#### 5.4.2. Thyroid Gland

From a 26-week oral toxicity study in rats, initial data on triiodothyronine (T3), thyroxine (T4), and thyroid stimulating hormone (TSH) are available [2,18]. T4 was decreased and TSH increased dose-dependently, predominantly in male rats and in some female rats, accompanied by changes in thyroid weight and hypertrophy of thyroid follicular cells (as summarized by [2,18]). In rhesus monkeys, sub-chronic oral exposure to CBD also significantly decreased relative thyroid weight [2,59].

Two recent OECD/GLP studies in rats administered oral doses of highly pure CBD from hemp isolate and also reported a decrease in T4 [44,45]. In one study, a hemp-derived CBD isolate (>95% purity, ethanolic extract of whole *C. sativa* L. plant, in medium-chain triglycerides) was administered orally (gavage) to rats at doses of 0, 30, 115, 230, and 460 mg/kg bw/day, with a 35-day recovery [45]. T4 levels were significantly reduced in females at and above a dose of 115 mg/kg bw/day and in males at and above a dose of 230 mg/kg bw/day, with T4 returning to control levels in the highest dose group by the end of an off-dose recovery phase of 35 days [45]. The second study was conducted as a reproductive/developmental toxicity screening study. Doses of 0, 30, 100, and 300 mg/kg bw/day of a hemp-derived CBD-isolate (>99% purity, in olive oil) were given via oral gavage to rats [44]. Minimal to moderate thyroid hyperplasia was observed after administration of 100 or 300 mg/kg bw/day CBD to male and female F0 rats [44]. Thyroid weights were not altered, but thyroid lesions were associated with significant decreases in serum T4 (males and females at 100 and 300 mg/kg bw/day, respectively) and T3 (females only at 300 mg/kg bw/day) concentrations [44]. Compared to the historical control values, however, only the T4 concentrations in the females of the high-dose group were lower [44].

In another study, a hemp-derived CBD-isolate (>99% purity, in olive oil) was administered via oral gavage to rats for 90 days (0, 50, 80, 120, or 140 mg CBD/kg bw/day), with a 28-day recovery phase [43]. The serum levels of T3 and T4 were unaltered by CBD treatment [43]. After administration of 80–140 mg CBD/kg bw/day, TSH levels increased significantly in male and female rats compared to the control group [43].

#### 5.4.3. Adrenal Glands

Based on a 26-week oral toxicity study with a 4-week recovery period, the EMA reported in its Epidyolex^®^ assessment report that the adrenal glands were target organs for toxicity in rats, in addition to the liver and thyroid, as evidenced by changes in organ weights [18]. It was noted that the mechanisms for adrenal toxicity are not known, but that vacuolization of the adrenal cortex can be triggered by various factors, e.g., a xenobiotic or drug affecting steroidogenesis in the adrenal cortex and/or the hypothalamic–pituitary–adrenocortical hormonal axis [18].

### 5.5. Reproductive System

In a 90-day oral toxicity study in rhesus monkeys, there were significant changes in organ weights in all dose groups of CBD in both sexes (0, 30, 100, or 300 mg/kg bw/day CBD (99% purity)) [59]. In addition, gonadal weight was lower in both genders, and testes size was 8–25% lower after 90 days. CBD-treatment-related alterations in reproductive systems, e.g., in testis weights of rats, were also documented. Developmental toxicity has been reported in the context of studies with rabbits and rats within the assessment of Epidyolex^®^ [18]. Treatment of rats with doses up to 250 mg CBD/kg bw/day from gestational day (GD) 6 to 17 resulted in more fetuses with a supernumerary liver lobe. Furthermore, a dose-related increase in the number of male offspring with reduced testes (at doses starting at 75 mg/kg bw/day) was seen. Fetal weights were reduced in rabbits after exposure to 125 mg/kg bw/day from GD7 to GD19. Currently, available data from animal studies point to the conclusion that CBD has a dose-dependent negative effect mainly on the male reproduction system [60,61]. However, in a recent OECD test guideline 421/GLP study in rats with a highly pure CBD hemp isolate, Henderson and colleagues reported unexpectedly high maternal toxicity (7/10 F0 females vs. 1/10 F0 males) at the highest tested dose of 300 mg/kg bw/day, along with adverse effects on the female reproductive tract and other organ systems, leaving only few pups for evaluation [44].

### 5.6. Fertility

There is some evidence that CBD affects sperm morphology and fertility. In an oral toxicity study in monkeys conducted by Rosenkrantz et al., inhibition of spermatogenesis was observed in almost all male monkeys treated with 30, 100, and 300 mg/kg bw/day. These observations were reflected by histological changes, such as smaller seminiferous tubules, lower mitotic index, fewer germ cells per tubule, and decreased numbers of spermatocytes, spermatids, and spermatozoa [59]. Decreased sperm quality and increases in sperm morphological abnormalities in mice treated with doses ≥ 15 mg/kg bw/day were also reported [60,61]. In addition, a significant reduction in fertility rates, as well as in impregnation rate, number of litters, and increases in prenatal losses were observed after sub-chronic oral exposure to 30 mg/kg bw/day CBD. Carvalho et al. also reported adverse effects in spermatogenesis and spermatozoa performance after oral CBD treatment in mice [62]. This included increased DNA damage and number of abnormal acrosomes in mature spermatozoa, as well as inhibition of enzymes that protect spermatozoa from oxidative stress. Gingrich et al. described the findings on spermatogenesis by Carvalho et al. as very similar to those reported in primates by Rosenkrantz et al., supporting the adverse effects of CBD on fertility [22]. Tallon and Child reported a statistically significant decrease in motility, epididymal sperm count, and homogenization-resistant spermatid (HRS) count from the testis of rats in the highest dose group after administration of a hemp-derived CBD isolate (>95% purity) at 30, 115, 230, and 460 mg/kg bw/day of CBD [45]. By contrast, there were no changes in sperm quality in rats in a 28-day study conducted according to OECD Test Guideline 421 by Henderson et al., which administered a hemp-derived CBD isolate (30, 100, or 300 mg/kg bw/day) via oral gavage to rats [44].

### 5.7. CBD Interaction with Drug Metabolism

CBD is extensively metabolized by CYP450s, which has been shown to affect the metabolism of several drugs [2]. CBD was reported to inhibit several human CYPs in in vitro incubations with human liver microsomes [63]. Based on these data, pharmacokinetic interactions between orally ingested CBD and drugs preferentially metabolized by CYP1A2/2C9/2C19/2D6/3A were predicted [2,63]. According to the US FDA, in vitro studies suggest that CBD inhibits CYP1A2, CYP2B6, CYP2C8, CYP2C19, and CYP3A4 (IC_50_ < 10 μM) but is also able to induce CYP1A2, CYP2B6, and CYP3A4 at clinically relevant concentrations [17]. In addition, the US FDA reported that CBD strongly inhibits UGT1A9 and UGT2B7 in human liver microsomes [17].

In mice, CBD resulted in induction of several hepatic CYP450s, e.g., CYP2B, 3A, and 2C, after i.p. administration of 120 mg/kg bw/day CBD for 4 days, or oral gavage administration of 0, 61.5, 184.5, or 615 mg/kg bw/day for 10 days [2,64,65]. However, EFSA noted that it is not clear whether these findings in mice are also relevant for humans [2].

According to EFSA, the interactions between CBD and neurological drugs used in the treatment of epilepsy have already been investigated, while data on potential interactions with other drugs that could also affect the kinetics of CBD are not available [2].

The UK Advisory Committee on Novel Foods and Processes (ACNFP) and the Committee on Toxicity (COT) pointed out that an inhibitory interaction has been observed at doses of 1 mg/kg body weight/day (equivalent to 70 mg in a 70 kg adult) and above when CBD is administered concomitantly with certain medications. Lower doses have not yet been studied [66].

### 5.8. Genotoxicity

Bacterial reverse mutation assays (Ames test) were negative for different CBD preparations [2]. Positive results with pure CBD (99.95%) were reported in non-guideline compliant in vitro studies for micronucleus induction in human HepG2 cells and in the comet assay using buccal-derived TR146 cells [84]. Unfortunately, no differentiation regarding aneugenicity and clastogenicity was made in this micronucleus test. In contrast to in vitro results, CBD was negative in an in vivo micronucleus test measuring micronuclei in bone marrow in male SD rats after oral gavage of the maximum tolerated dose (MTD) of 500 mg/kg bw/day in a sesame oil vehicle and an in vivo comet assay measuring liver DNA damage in male SD rats after a single oral MTD dose of 500 mg/kg bw/day [18,67]. Both tests were performed according to ICH S2 (R1) (scientific guideline on genotoxicity testing and data interpretation for pharmaceuticals intended for human use) and under GLP, although documentation of this is limited (e.g., inconclusive test item identity and purity). Recently, the potential genotoxicity of a pure CBD isolate was assessed by Henderson et al. using in vitro and in vivo assays following OECD testing guidelines and performed according GLP [68]. Pure CBD (>99%, produced under current GMP by ethanol extraction and subsequent crystallization) up to 5000 µg/plate was negative in the Ames test (OECD 471), with or without metabolic activation. These results confirmed earlier investigations in the Ames test. In addition, 6, 10, or 11 µg/mL CBD was also negative in an in vitro micronucleus assay (OECD 487) using TK6 cells with metabolic activation (4 h exposure time), and without metabolic activation (4- and 27-h exposure time). Furthermore, CBD up to 1000 mg/kg bw/day in olive oil (MTD) was negative in an in vivo micronucleus assay (measuring micronuclei in bone marrow after two treatments by oral gavage) in male and female SD rats (OECD 474). A bioanalysis (plasma) confirmed the exposure of the target tissue (bone marrow/erythroblasts). Mutagenic, clastogenic, as well as aneugenic effects of CBD can therefore be ruled out based on the studies of Henderson and colleagues.

In summary, the Ames tests, in vivo micronucleus assays and the in vivo comet assay were negative, indicating no systemic genotoxic hazard. However, the available data may not rule out a potential concern regarding clastogenic or aneugenic effects at the site of first contact [2].

## 6. Characterization of Beneficial Health Effects of CBD

Health claims considered in the present assessment were selected based on the most frequent mentions on industry websites and health portals, and include “Sports nutrition: during exercise improves/enhances endurance performance/perform for longer/can help to reduce exercise induced muscle pain/can protect muscle during exercise”, “Helps to maintain the cardiovascular system/heart health”, “Helps to maintain an optimal relaxation/helps to support the relaxation”, “Helps to maintain a healthy sleep/contributes to the reduction of time taken to fall asleep”, “Helps maintain positive mood and good cognitive functions/neuroprotective”, “Contributes to the normal function of the immune system/ensures activity of the immune system/reduction of inflammatory reactions”, “Contributes to the protection of cells from oxidative stress/improves the body’s resistance to stress/helps the body to deal with stress”, “Reduction of menstrual discomfort”, “Helps to moderate signs of anxiety”, “Pain relief”.

### 6.1. Physical Performance

Based on the assumption that reduction of muscle damage and inflammatory processes after training is beneficial, the prevalence of foods/food supplements with anti-inflammatory and antioxidant effects is increasing. As a result, interest in CBD products has been increasing, not only in recreational but also in competitive sports [85,86,87,88]. Since 2018, the World Anti-Doping Agency (WADA) has taken CBD off the banned list, making CBD the only cannabinoid that may be used in competitive sports. This is mainly because CBD has no psychotropic effect, which has been shown in a large number of human studies even with very high doses of up to 6000 mg/day [54].

Currently, only a small number of studies evaluating the influence of *Cannabis*, and particularly CBD, on physical performance in humans is available (Table 2). Only one study was conducted in the context of endurance performance [89]. In a placebo-controlled double-blind study with crossover design, Sahinovic et al. compared the influence of 300 mg CBD (4.3 mg/kg bw/day for a 70 kg person; application: 90 min before the test) on physiological and psychological factors after 60 min of running at 70% of the maximum rate of oxygen consumption (VO2max). Although significant effects of CBD on oxygen uptake and subjective well-being were demonstrated, the authors noted that the findings lacked robustness due to the relatively small effect size and the limited sample size. They were also unable to determine any influence of either the exercise protocol or CBD application on serum concentrations of the pro-inflammatory biomarkers, interleukin-6 (IL-6), and tumor necrosis factor α (TNF-α) [89].

Regarding aerobic fitness training, a recent study examined the impact of CBD on several aspects, including health-related fitness, physical activity, cognitive health, psychological wellbeing, and levels of C-reactive protein (CRP) in healthy individuals [90]. Eight weeks of calorie-matched placebo or oral capsules containing 50 mg of CBD (one capsule per person; 0.7 mg CBD/kg bw/day) were given to the forty-eight participants who were randomly assigned to two groups. Participants underwent pre- and post-intervention assessments, including blood and body composition analysis, fitness evaluation, physical activity measurements, and self-reported questionnaires. No significant differences were observed between both groups regarding body composition, aerobic fitness, muscular strength, physical activity, cognitive health, psychological wellbeing, and resting CRP levels. In contrast to the CBD group, the placebo group experienced a decline in mean and relative peak power after 8 weeks. The authors concluded that CBD supplementation for 8 weeks may prevent reductions in anaerobic fitness over time. Nevertheless, CBD supplementation for several weeks may not be enough to change measures related with health-related fitness, mental health, and inflammation in healthy subjects.

Other studies have been conducted regarding CBD and strength training. In a controlled, single-blinded study with a parallel group design [91], 23 healthy subjects performed an intensive squat protocol with 4 sets of 10 repetitions at 80% of one-repetition maximum. Subsequently, one group consumed 16 mg CBD (0.24 mg/kg bw/day) and one group consumed a placebo preparation. The third group was not administered any supplements (null group). A visual analogue scale (VAS) was used to determine subjective fatigue before, immediately after, and 24, 48, 72, and 96 h after exercise. The authors found that the CBD group experienced the greatest increase in subjective fatigue and a significant decrease 24 h after exercise. However, the performance level of the CBD group was significantly higher at the beginning than the other groups. Another study [92] investigated the daily intake of CBD (150 mg/day for 3 days, divided into two doses of 75 mg each; corresponding to ~2 mg/kg bw/day) during the recovery process after eccentric training. In a double-blinded crossover study, 13 untrained men completed 6 sets of 10 isokinetic-eccentric biceps curls. In addition, the maximum torque during an isometric biceps curl with a joint angle of 115°, the subjective feeling of fatigue, the elbow angle of the relaxed, hanging arm, and the arm circumference were measured before and 24, 48 and 72 h after the exercise. Significant changes due to the training were only found in subjective fatigue perception and joint angle. The other parameters showed no change due to training, and no effects of CBD could be determined. Crossland and colleagues also investigated the influence of CBD on strength capacity and muscle damage [93]. In a randomized controlled double-blind crossover study, 24 female athletes performed 10 sets of 10 repetitions of isokinetic eccentric leg extensions (30″/s) twice 30 days apart, with one-minute rest between sets. At baseline and 4, 24, and 48 h after exercise, vertical jump, isometric, and dynamic (60, 180, and 300″/s) maximal leg extension strength were measured. In addition, myoglobin, interleukin-1β (IL-6), and interleukin-10 (IL-10) concentrations were determined at the same timepoints. Two hours before, immediately after, and 10 h after the fatigue protocol, subjects consumed either 5 mg/kg bw CBD (224–408 mg per application) or a placebo preparation. At no time was there a significant increase in inflammatory markers or a difference between placebo and CBD. Myoglobin was significantly increased in both groups after 4 h, but not after 24 and 48 h. A group difference could not be found. Isometric and dynamic maximum strength (60″/s) were significantly reduced after 4 and 24 h without any difference between the groups. In contrast, significant effects of 60 mg CBD (~0.86 mg/kg bw/day) on muscle damage were observed in studies by Isenmann and colleagues [94,95]. In a randomized controlled pilot study with a crossover design and very well-trained athletes (squat performance > 150% of bw), significantly lower creatine kinase (CK) concentrations were found 24 h after exercise following intensive strength training with 3 sets of 12 repetitions of an intensity of 70% of one-repetition maximum followed by 3 sets of 15 low jumps [95]. However, strength capacity was lower after CBD administration, compared to placebo. In contrast, in well-trained athletes (squat performance: 120–150% of bw), significant differences were found only 72 h after exercise with the same training protocol. In a six-arm randomized controlled crossover study, the influence of a single 60 mg CBD application on muscle damage and strength capacity 24, 48, and 72 h after exercise was examined [94]. Based on the different results of the available studies, the performance level as well as the training protocol probably play a decisive role in increasing muscle damage, inflammatory reactions, the reduction of performance, and the influence of CBD application.

In a randomized, double-blind, placebo-controlled, repeated-dose pilot study, Peters et al. investigated the effects of CBD and cannabigerol (CBG) on the recovery from delayed onset muscle soreness (DOMS) [96]. Exercise-trained individuals underwent an experimental induction of DOMS and completed follow-up examinations 24, 48, and 72 h post- DOMS. During this time, they consumed twice daily formulations containing CBD and CBG, along with branched-chain amino acids (BCAAs), beta caryophyllene (BCP), and magnesium citrate. The overall daily intake of the active formulation consisted of 70 mg CBD (~1 mg CBD/kg bw/day), 100 mg CBG, 50 mg BCP, 7.6 g BCAAs, and 840 mg magnesium citrate. The first dose of the study formulation was administered one hour prior to undergoing the DOMS induction. The formulation did not exert meaningful effects on objective measures of recovery, although it showed minor effects on decreased self-reported average soreness/discomfort 72 h post-DOMS. The authors stated that the observed effects were preliminary and should be interpreted with caution.

A recent study conducted by Isenmann et al. examined the effect of a 6-day chronic oral CBD administration on muscle recovery and performance following an intensive training protocol [97]. The effects of two different CBD products on performance, muscle damage, and inflammation in well-trained athletes were assessed in a three-arm, double-blind crossover study. Seventeen participants completed a six-day, high-intensity training regimen on three occasions, after each of which each subject took either a placebo or a CBD product (60 mg of oil or solubilisate; ~0.86 mg CBD/kg bw/day). The training protocol was able to induce muscle damage and a performance decrease. Only the oil formulation was associated with a reduction in myoglobin concentration (*p* < 0.05) in experienced athletes. Regarding immune function, a significant reduction in platelets lymphocyte ratios was noted in well-trained athletes following placebo administration (*p* < 0.05). CBD oil had a minor inhibitory effect (*p* < 0.10). None of the CBD products had an impact on physical performance or inflammatory parameters.

Recently published reviews concluded that CBD and Δ^9^-THC do not enhance performance, based on the data by Kennedy et al. [98]. According to Burr et al., further research is needed to elucidate the dose–response effects of CBD, taking into account consumption methods, timing around exercise, and cannabinoid concentrations [99]. Athletes’ consumption of CBD is probably more important to recovery during both training and competition (e.g., to reduce muscle damage and/or facilitate post-exercise recovery). CBD may have some potential for enhancing pain relief and recovery in athletes via several possible mechanisms, although there is currently limited evidence supporting these potential beneficial effects.

In summary, there is currently no convincing evidence for beneficial effects of CBD at doses below 300 mg/day on recovery and performance.

### 6.2. Cardiovascular System

There is increasing evidence supporting the role of the endocannabinoid system (ECS) in cardiovascular functions (as reviewed in [100]). An altered expression of cannabinoid (CB) receptors and endocannabinoid levels leads to an increased activity of the ECS and is observed particularly in stress and inflammatory situations [100]. ECS overactivity has been reported to be associated with multiple pathophysiological conditions, including cardiovascular disease [100]. Different effects are attributed to CB receptor activation, depending on the receptor type. Karimian Azari et al. reported that CB1 receptor activation has been associated with cardiac dysfunction, whereas activation of the CB2 receptor may afford cardiovascular protection [100].

CBD oils are being increasingly marketed with health claims related to improved cardiovascular health. The literature search revealed that many of the available studies investigating cardiovascular endpoints were conducted under pathological conditions, e.g., in patients addressing therapeutic use of CBD or in various animal models of cardiovascular disease (hypertension, stroke/stress, myopathy, myocarditis, obesity, diabetes). Thus, only a few studies addressing the effects of CBD on the cardiovascular system in healthy individuals are available, and in most studies only a single dose was administered (Table 3). There were few studies on chronic or repeated administration, and only four studies investigated doses in the range of the therapeutic starting dose or below (≤300 mg CBD/person/day; ~4.3 mg/kg bw/day).

In a study conducted by Cunha et al. [101], 210 mg CBD/day (~3 mg/kg bw/day) or placebo was given for 30 days to 16 healthy human volunteers. No influence on heart rate and electrocardiogram (ECG) was observed. In a second study conducted by this group, CBD at a dose of 200–300 mg per person (~2.9–4.3 mg/kg bw/day) was administered for 4.5 months to patients with epilepsy [101]. Again, no influence of CBD on heart rate and ECG was observed. In a preliminary study, five patients with neurological movement disorders received increasing oral doses of CBD from 100 to 600 mg/day (~1.4–8.6 mg/kg bw/day) over 6 weeks [102]. Hypotensive effects of CBD were observed in some patients, but only at higher therapeutic doses of 300–400 mg CBD/day (corresponding to approx. 5–7 mg/kg bw/day). A randomized, placebo-controlled, triple-blinded (participant, investigator, outcomes assessor), crossover trial examined the effect of five weeks of CBD administration on 24 h blood pressure in individuals with mild or moderate hypertension, who were either untreated or receiving standard therapy [103]. CBD was applied in the form of a patented formulation to increase bioavailability, with 225–300 mg (~3.2–4.3 mg/kg bw/day) split into three daily doses for the initial 2.5 weeks and 375–450 mg (~5.4–6.4 mg/kg bw/day) split into three daily doses for the following 2.5 weeks. Administration of CBD significantly reduced average 24 h mean, systolic, and diastolic blood pressure after 2.5 weeks; however, these values largely remained stable after increasing the CBD dose [103]. There was no significant difference in pulse wave velocity at different time points, regardless of the intervention arm [103]. No changes in liver enzymes or serious adverse events were observed [103]. A sub-study on potential mechanisms reported that CBD supplementation appears to reduce arterial blood pressure via modulation of the sympatho-chromaffin system [104]. Whether CBD affects the cardiovascular system in humans also seems to depend on the method of administration and the preparation of CBD. A single dose of 90 mg CBD (~1.3 mg/kg bw/day) applied orally did not influence heart rate, blood pressure, and cerebral blood flow, whereas the same dose of CBD encapsulated in a patented capsule formulation (claimed to improve CBD bioavailability) increased circulating CBD levels, decreased diastolic blood pressure, and increased cerebral blood flow [105]. Zuardi et al. investigated whether the application of a single oral dose of 300 mg of CBD (~4.3 mg/kg bw/day) to healthy volunteers had an influence on the cardiovascular system [106]. No changes in blood pressure and heart rate were observed. A limitation of the available studies is that the CBD source and purity level were often not reported (see Table 3). No further studies in the dose range ≤ 5 mg/kg bw/day were identified. Thus, the few and preliminary studies conducted at or below the therapeutic starting dose of 5 mg/kg bw/day mostly showed no effect of CBD on cardiovascular parameters. In one study, beneficial effects on blood pressure were observed only in the range of the therapeutic starting dose and above [102].

To assess whether CBD at doses > 5 mg/kg bw/day (>300 mg CBD/person) could positively affect the cardiovascular system, studies in this therapeutic dose range are also briefly summarized here. Studies in which CBD doses were administered repeatedly (from 7 days up to 6 weeks) show inconsistent results. Some studies reported a reduction in blood pressure after ingestion of CBD at doses of 600 mg CBD/day (~8.6 mg/kg bw/day), especially under stressful conditions [107,108], while other studies found no effect at doses of 600–1200 mg CBD/day (~8.6–17.1 mg/kg bw/day) [109,110,111]. In the majority of studies in which a single therapeutic dose of CBD (approx. 600 mg/day; ~8.6 mg/kg bw/day) was administered, there was no effect of CBD on cardiovascular parameters such as blood pressure or heart rate ([112,113,114,115]; for further studies, see [116,117]). The objective of most of these studies was to evaluate and compare the acute pharmacological or other symptomatic effects of CBD with those of Δ^9^-THC. A series of studies also assessed whether a single oral pre-treatment with 600 mg of CBD (~8.6 mg/kg bw/day) can prevent the acute psychotic symptoms induced by Δ^9^-THC or Cannabis or can attenuate the behavioral effects of the N-methyl-D-aspartate (NMDA) receptor antagonist, ketamine. In all these studies, treatment with a single CBD dose had no influence on either heart rate or blood pressure [118,119,120,121]. Most of the studies examined various primary outcomes unrelated to cardiovascular parameters but monitored heart rate and blood pressure as secondary outcomes.

Animal studies also showed inconsistent results regarding the effect of CBD on cardiovascular parameters. It should be noted that in the available animal studies, CBD was mostly administered i.p. or i.v., whereas virtually no studies are available for oral administration. Repeated i.p. administration of 5–10 mg CBD/kg bw/day for 2–4 weeks showed no influence of CBD on biochemical parameters in cardiac tissues and cardiac histopathology in rats exposed to doxorubicin [122], or on blood pressure and heart rate in hypertensive rats [123], while cardiomyocyte width in the left ventricle and vasoconstriction of coronary arteries were reported to be reduced in hypertensive rats upon CBD exposure [124]. After a single i.v. or i.p. administration, CBD showed positive effects on blood pressure and heart rate in some studies [125,126,127]. In other studies, blood pressure and heart rate were increased [128,129] or were not affected [129,130]; for details of the studies, see [116]. In studies using animal models of cardiovascular or other diseases, the effects of CBD in the therapeutic dose range of approx. 5–20 mg/kg bw/day on cardiovascular parameters were inconsistent and varied depending on the model used. Some studies showed no impact of CBD administration, but the majority showed positive effects (summarized by [100,116]).

In in vitro studies and ex vivo models, effects of CBD on cardiovascular endpoints (e.g., vasodilation of isolated vessels or perfused hearts) were observed in the µM concentration range (1–1000 µM) (reviewed by [100]; e.g., [131,132,133,134]).

**Table 3:** The impact of CBD on the cardiovascular system.

Study Design		CBD Dose *		Source		Route		Duration		Effects		Comment/Limitations		Reference
**Repeated dosing**														
Healthy human volunteers (*n* = 8/group); double-blind, placebo-controlled		210 mg/day (3 mg/kg bw/day) or placebo		CBD isolated from hashish of undetermined age		Oral		30 days		No influence on heart rate and ECG was observed.				[101]
Patients with epilepsy (*n* = 15); randomized, double-blind, placebo-controlled		200–300 mg/day (2.9–4.3 mg/kg bw/day)		CBD isolated from hashish of undetermined age		Oral		4.5 months		No influence on heart rate and ECG was observed.		Patients also received antiepileptic drugs		[101]
Patients with dystonic movement disorders (*n* = 5); preliminary open study		Oral doses of CBD rising from 100 to 600 mg/day (1.4–8.6 mg/kg bw/day)				Oral		6 weeks		Hypotensive effects of CBD have been observed in some patients, but only at higher doses of 300–400 mg CBD/day (~5–7 mg/kg bw).		Patients also received standard medication for dystonic movement disorders		[102]
Individuals with mild or moderate hypertension (*n* = 70); either untreated or receiving standard care therapy; randomized, placebo-controlled, triple-blind, crossover design		225 to 300 mg split into three daily doses for the initial 2.5 weeks (3.2–4.3 mg/kg bw/day) and 375 to 450 mg split into three daily doses for the following 2.5 weeks (5.4–6.4 mg/kg bw/day)		DehydraTECH^TM^ 2.0 CBD, a patented formulation with increased CBD bioavailability		Oral		5 weeks		Administration of CBD significantly reduced average 24 h MBP, SBP, and DBP after 2.5 weeks. Values largely remained stable after increasing the CBD dose. No changes in liver enzymes or serious adverse events No significant difference in pulse wave velocity at different time points		HYPER-H21-4 trial; study design described by Kumric et al., 2022		[103]
**Single administration**														
Healthy young men (*n* = 13); double-blinded, placebo-controlled, crossover design		45, 90 mg/day; (0.6, 1.3 mg/kg bw/day)		CBD capsules; 45/90 mg CBD; 150/300 mg organic multi-spectrum hemp oil		Oral		Acute		No influence on BP, HR, and CBF				[105]
		45, 90 mg/day (0.6, 1.3 mg/kg bw/day)		CBD encapsulated as TurboCBDTM; 45/90 mg CBD; 600/1200 mg American ginseng; 240/480 mg ginkgo biloba; 150/300 mg organic hemp oil		Oral		Acute		Increased circulating CBD levels Tendency for decreased DBP and MBP Increased CBF		Patented capsule formulation increasing CBD bioavailability A potential influence of the combined ginkgo biloba, ginseng, and hemp oil should be considered.		
Healthy volunteers (*n* = 40, 10/group); double-blind, placebo-controlled; 4 groups: placebo, CBD, diazepam (10 mg) or ipsapirone (5 mg)		300 mg/day (4.3 mg/kg bw/day)		Not specified		Oral		acute		No changes on BP, HR				[106]

* Calculated for a person with 70 kg bw.; BP: blood pressure; CBD: cannabidiol; CBF: cerebral blood flow; DBP: diastolic blood pressure; ECG: electrocardiogram; HR: heart rate; MBP: mean blood pressure; SBP: systolic blood pressure.

Kicman and Toczek reviewed the effects of CBD on the cardiovascular system in health and disease [116]. They concluded that studies carried out in animals and humans under physiological conditions largely indicate no or little effect of CBD administered orally, i.v., intra-arterially, i.p., or via inhalation (after acute and repeated dosing) on systolic, diastolic, or mean arterial blood pressure and/or heart rate [116]. By contrast, under experimental pathological conditions, such as, e.g., hypertension, heart diseases, stroke, diabetes, or hepatic and renal ischemia/reperfusion injury, often a protective effect of CBD was reported [116]. Results of a meta-analysis (including 25 studies) by Sultan et al. indicated no influence of CBD on heart rate and blood pressure after acute and chronic CBD administration under physiological conditions, but a reduction of blood pressure and heart rate under stressful conditions [117].

Overall, it can be concluded from the available data that acute and repeated administration of CBD to healthy human subjects at doses below 5 mg/kg bw/day (300 mg/person/day) and in the higher therapeutic dose range at or above 5 mg/kg bw/day showed no effects on blood pressure, heart rate, or blood pressure volume under controlled/physiological conditions. Under stressful conditions, some studies reported a reduction in blood pressure after ingestion of CBD at therapeutic doses ≥ 5 mg CBD/kg bw/day. Animal studies conducted under physiological conditions mainly used CBD doses in the therapeutic dose range and reported inconsistent results, but the majority observed no influence on blood pressure and heart rate. In studies with animal models of cardiovascular or other diseases, CBD was also mainly administered in the higher therapeutic dose range. Effects on cardiovascular parameters were inconsistent and varied depending on the model used but mostly showed positive effects. However, there was considerable heterogeneity in terms of animal species and model, dose and route of administration, and method and timing of end point measurement, making it difficult to compare studies.

In conclusion, there is currently no convincing evidence that CBD positively influences the cardiovascular system in the dose range below the therapeutic starting dose of 300 mg/person/day or 5 mg/kg bw/day. It should be noted that human studies in this dose range are limited and often preliminary.

### 6.3. Immune System

Phytocannabinoids are believed to possess a wide range of immune regulatory properties, mediated by the endocannabinoid system. There are plenty of data showing immunosuppressive and anti-inflammatory effects of CBD in animal models and cell culture systems. Nichols et al. summarized a variety of these studies and data in a review [135].

#### 6.3.1. Immune Suppression

There is mechanistic evidence based on in vivo and in vitro data indicating that CBD is immunosuppressive [135]. The involved mechanisms include direct prevention of activation of different immune cell types, promotion of regulatory cells, which control other immune cell targets, and induction of apoptosis [135]. However, only a few human studies examining the activities of CBD on the immune system are available (Table 4). Even for the approved CBD-based drug Epidyolex^®^ there seems to be a lack of evidence regarding the long-term safety and efficacy with regard to the immune system [136]. Rachayon et al. examined the effects of CBD on the inflammatory response system, the chronic inflammatory response system, M1 macrophages, T helper (Th)-1, Th-2, Th-17, T regulatory (Treg) profiles, and growth factors in patients with depression and healthy subjects [137]. In their study, the supernatant of stimulated whole blood (25 μg/mL of lypopolysaccharid (LPS), 5 μg/mL of phytohemagglutinin (PHA)) of 30 patients with depression and 20 control subjects was assayed for cytokines in the absence and presence of three CBD concentrations (0.1, 1, and 10 µg/mL). The results indicated no beneficial effects of CBD on the stimulated immune profile of depression. The authors also speculated that higher concentrations of CBD might exacerbate inflammatory processes.

Hobbs et al. carried out a randomized, double-blind, pilot study with parallel arm design in 10 healthy adults to assess the differences in pharmacokinetics of purchasable lipid- and water-soluble powder formulations of CBD and to analyze their potential acute anti-inflammatory activity [138]. Participants consumed a single 30 mg dose (0.43 mg/kg bw), which is within the range of typical commercial supplement doses. Blood samples were collected over 6 h and cell supernatants were assayed for the inflammation markers IL-10 and TNF. TNF was decreased in LPS-stimulated peripheral blood mononuclear cells (PBMC) collected 90 min after CBD exposure relative to cells collected at baseline.

Flores et al. investigated the effects of CBD administration on cognitive health, psychological wellbeing, physical activity, CRP levels, and health-related fitness during eight weeks in healthy individuals, without identifying any significant differences upon CBD administration (see also Section 6.1) [90].

In conclusion, some evidence for an immunosuppressive activity of CBD is available in cell culture and animal models; however, there is a general consensus that clinical results are still lacking [139].

#### 6.3.2. Antioxidative Activity

Immune reactions may cause the formation of reactive oxygen species (ROS), which can lead to tissue and organ damage. CBD was reported to have high antioxidant activity and to control the formation of ROS, which is supposed to influence the progression of certain diseases (based on reports reviewed by [140,141,142]). Numerous in vitro studies indicated that CBD alters the abundance and activity of antioxidant molecules. The most important targets are redox-sensitive transcription factors such as Nrf2, which initiate the transcription of antioxidant genes, e.g., the expression of inducible antioxidant enzymes that regulate cellular ROS levels (in vitro studies reviewed by [142]). There are indications that CBD regulates the activity of superoxide dismutase (SOD), which metabolizes superoxide radicals. CBD is proposed to affect the redox balance and free radical scavenging by influencing intrinsic mechanisms (based on in vitro studies reviewed by [142]). However, pro-oxidant activities of CBD have also been described, which may depend on the in vitro cell systems and the CBD concentrations used [142].

There are no human studies regarding the effects of CBD on oxidative stress parameters. In animal studies, CBD was mostly administered i.p., whereas no studies are available for oral administration. In rats, administration of CBD (i.p. 2.5, 5, 10 mg/kg bw immediately after induction of sepsis by cecal ligation and perforation (CLP)) changed oxidative stress parameters in the brain and peripheral organs [143]. In ischemic rats, injection of 50, 100, and 200 ng/rat CBD into the right lateral ventricle of the brain increased the activity of endogenous antioxidant enzymes such as SOD and catalase in the brain [144]. In exhaustive exercise training mice, CBD (i.p. 50 mg/kg bw) showed protective effects on myocardial injury, which is thought to also be induced by oxidative stress [145]. In mice with induced oral mucositis induced by 5-fluorouracil, antioxidant enzyme activity was slightly reduced by i.p. injection of CBD (3, 10, and 30 mg/kg bw) [146]. In a mouse model for hepatic ischemia-reperfusion injury (induced by midline laparotomy incision of the exposed liver), i.p. injection of 3 and 10 mg/kg bw CBD decreased oxidative stress parameters in the liver [147]. In diabetic mice, parameters of myocardial oxidative stress were reduced by i.p. injection of 1, 10, or 20 mg/kg bw CBD. In the hearts of diabetic mice, there was an increased accumulation of lipid peroxides, protein carbonyls, ROS generation, expression of mRNA of various ROS generating NADPH oxidases, with concordant decreases in the glutathione/glutathione disulfide (GSH/GSSG) ratio and decreased SOD activity. These changes were attenuated when mice were treated with CBD for 11 weeks [148].

In conclusion, no human studies are available investigating effects of CBD on parameters of oxidative stress. A relationship between the oral intake of CBD at doses below 300 mg/day and the reduction of oxidative stress has not been demonstrated in animal models.

### 6.4. Nervous System

#### 6.4.1. Positive Mood

Acute use of Cannabis is frequently associated with positive/good mood, primarily linked to the psychotropic effects of Δ^9^-THC. Currently, many CBD products including oils, sweets, mouth sprays, capsules, or sublingual drops are commonly advertised with claims of mood improvement/boosting. In most cases, the formulation of such products is unclear or unspecified, and aside from CBD and other cannabinoids, these products often contain the psychotropic component Δ^9^-THC.

Some studies investigated whether CBD has the potential of reversing Δ^9^-THC/cannabis-induced effects (e.g., effects on cognition, impairment of emotions, psychosis-like effects), or even behavioral effects caused by alcohol consumption [149,150,151]. While certain studies support the capacity of CBD to mitigate some of the effects of Δ^9^-THC (e.g., psychotomimetic effects and effects on memory, cognition or psychomotor performance), others suggest that CBD may influence and even increase some of its effects [152,153,154]. Studies investigating the use of Cannabis for mood/psychiatric disorders, such as major depressive disorder, anxiety, post-traumatic stress syndrome, bipolar disorder, or epilepsy are not included here. Similarly, studies evaluating the effects upon administration of CBD together with Δ^9^-THC (i.e., products containing a mixture of CBD and Δ^9^-THC) or high-THC Cannabis are also excluded.

A few studies researching other aspects (e.g., pain) have also reported mood changes as a secondary outcome (Table 5). For instance, the analgesic effects, safety, and tolerability of acute CBD administration (200, 400, 800 mg/person; 2.9, 5.7, 11.4 mg/kg bw/day) has been investigated in healthy volunteers, whereby CBD had subtle, dose-dependent subjective effects on mood [56]. However, this study presented several limitations, such as the source and frequency of CBD administration being poorly described. Additionally, mood records were based on self-perceived rating of mood and physical symptoms. Lopez et al. assessed several aspects of affect, mood, sleep and mental state after administration of CBD-containing hemp oil extract in healthy subjects but found no statistically significant changes using visual analogue scales [155]. Arndt and de Wit studied the response to emotional stimuli in healthy adults following CBD administration and found no subjective effects or changes in mood and anxiety [156]. Other studies evaluating the safety and tolerability of CBD also assessed aspects related to mood and psychotic symptoms but found no effects of CBD even when administered at high doses (i.e., 600 mg/day; 8.6 mg/kg bw/day) [112]. Overall and compounded by the fact that maintaining a “positive mood” is a vague concept as a health claim, there is no scientific evidence supporting any effects of CBD at the dose range considered here (<300 mg/day; <4.3 mg/kg bw/day) on the maintenance of a positive mood in healthy subjects.

#### 6.4.2. Good Cognitive Functions

Several studies have focused on the potential of CBD to improve the cognitive impairment (particularly memory and emotional processing) caused by Cannabis use (i.e., acute use of Δ^9^-THC) (reviewed in [157]). Some articles reported that pre-dosing with CBD or Cannabis containing high CBD content may protect against some Δ^9^-THC-induced verbal learning and memory deficits (reviewed in [158]). Studies evaluating CBD’s effects on cognition in Cannabis-using subjects are, however, out of the scope of this review and are therefore not further detailed here.

Although the effects of CBD (and Δ^9^-THC) on human cognitive functions (verbal memory, attention, emotional, visual, and/or auditory processing) have also been studied in healthy subjects, the most frequently tested dose was 600 mg (8.6 mg/kg bw/day), and the studies only evaluated acute/single CBD administration. Most of these studies reported opposing effects of Δ^9^-THC and CBD on brain activity related to at least some cognitive functions, but there were no significant differences following CBD administration that indicated an improvement in cognitive function [114,119,159,160]; for a review, see [157]. In a placebo-controlled, double-blind trial with 60 healthy volunteers, even higher doses (800 mg; 11.4 mg/kg bw/day) of CBD did not influence working memory, cognitive processing speed, attention, or emotional state [161].

To date, very few studies have assessed the effect of CBD alone (i.e., without other cannabinoids) on cognitive functions (Table 5). McCartney et al. evaluated cognitive performance after acute administration of 15, 300, or 1500 mg CBD (0.2, 4.3, 21.4 mg/kg bw/day) in healthy subjects by examining its effect on everyday tasks such as car driving performance [162]. Although these studies primarily focused on aspects other than assessing any potential cognitive improvement of CBD (i.e., proving CBD safety), the authors concluded that CBD administration is unlikely to impair cognitive function, without reporting any positive cognitive effects following CBD administration. Similarly, a study investigating the effects of oral administration of 50 mg/day CBD (0.7 mg/kg bw/day) for 8 weeks on fitness, psychological well-being and cognitive health showed no improvement on any assessed aspects relative to cognition [90].

A systematic review by Batalla et al. summarized current literature on the effects of CBD on brain function during resting state and performance of cognitive tasks. Although overall CBD impacts brain activity, the reviewed studies used high doses of CBD (sometimes in conjunction with Δ^9^-THC) and evaluated only effects after acute administration via different routes of administration [163]. Bloomfield et al. observed increased cerebral blood flow in the hippocampus, a region involved in memory processing, after oral, acute administration to healthy volunteers of 600 mg CBD (8.6 mg/kg bw/day) at least two times separated by a week [164]. These results, however, contrast with previous studies, which showed a decreased cerebral blood flow in both healthy and anxiety disorder participants [57]. Moreover, the authors noted that the reported effects may not translate to those observed after repeated CBD dosing and did not report any differences in memory performance following CBD administration.

In conclusion, there is no evidence that oral administration of CBD contributes to the maintenance of good cognitive functions in healthy subjects in the dose range below 300 mg/day (4.3 mg/kg bw/day), despite the fact that products continue to be advertised with claims of neurogenesis promotion, memory enhancement, and focus improvement for diseases such as attention deficit hyperactivity disorder (ADHD).

#### 6.4.3. Neuroprotection

CBD has long been mentioned as a promising drug for neurological disorders due to its claimed neuroprotective effects (reviewed by [165,166]). Some studies evaluated the outcome of CBD administration in the dose range < 300 mg/day (4.3 mg/kg bw/day) in relation to Cannabis use withdrawal or cigarette smoking, suggesting a potential restorative effect of CBD after brain structural damage conferred by chronic Cannabis use [167]. Other studies investigated the potential of CBD to improve the impairment of cognitive functions and attention, or the alteration of emotional experience induced by the psychotropic cannabinoid Δ^9^-THC, yielding conflicting results [161]. Several studies reported that CBD can promote neurogenesis, improve memory, and enhance focus. Additionally, some authors showed that CBD may be neuroprotective, based on its antioxidant and anti-inflammatory properties, suggesting that it could afford protection against numerous neuropathological disorders (reviewed in [168,169,170]).

Due to the ability of CBD to modulate the endogenous cannabinoid system, there has been an increasing interest in CBD as a potential alternative treatment for several brain disorders defined by neuronal loss and/or damage. As such, the literature search on neuroprotective effects of CBD mostly provided results regarding animal models of neurodegenerative diseases, such as Alzheimer’s (reviewed by [171]), Huntington’s [172,173], and Parkinson’s disease ([174,175]; reviewed by [171,176]) as well as in experimental animal models of hypoxic-ischemia [177] and encephalopathy [178,179] or studies with patients with the above-mentioned diseases [110,180,181,182] reviewed in [183,184]. In addition, various in vitro studies assessed CBD effects after induction of damage mimicking various neurological diseases, some of them suggesting that CBD is able to prevent a number of the cellular and molecular alterations associated with neurological damage [185,186]. Although some of these studies point towards a beneficial effect of CBD in multiple processes impaired in neurological diseases, studies with patients are still limited and there is no conclusive evidence that the observed effects in vitro and in animal models would translate to humans. More importantly, there was no evidence supporting a neuroprotective effect of CBD in the low dose range (<5 mg/kg bw/day) in healthy subjects.

Overall, there is currently no conclusive evidence demonstrating a beneficial effect of chronic administration of CBD at doses below the therapeutic starting dose (<300 mg/day) on maintaining a positive mood and maintaining good cognitive functions nor of a neuroprotective effect in healthy subjects. While good/positive mood is an aspect difficult to adequately characterize in scientific studies, the available literature does not support a beneficial effect of CBD in healthy subjects regarding this endpoint. Moreover, the literature pertaining cognitive effects of CBD administration mainly focused on evaluating its safety in comparison to Δ^9^-THC, rather than assessing any potential favorable effect. Although a few studies showed increased cerebral blood flow in the hippocampus, a region involved in memory processing, after administration of high doses of CBD (600 mg CBD), the discrepancies and limitations of the studies make it difficult to draw clear conclusions on the presumed positive effects of CBD on cognitive function. Furthermore, CBD has been linked to neuroprotection, mostly due to its anti-inflammatory and antioxidant properties, as well as its capacity to modulate autophagy and enhance neuronal survival, which could improve symptoms associated with neurodegeneration [183,187]. Although some research indicated neuroprotective effects and suggested that chronic CBD use could be beneficial for neurodegenerative diseases, evidence remains limited. The beneficial effects attributed to the use of CBD are often inconsistent among different studies. Conclusions are often limited due to the shortcomings of the studies, including the low number of subjects, heterogeneity of the product formulation tested (which influences CBD bioavailability), and differences in the route of administration, which affects CBD pharmacokinetics. Additionally, there are few studies in healthy subjects, and the long-term effects of CBD administration remain to be addressed.

**Table 5:** The impact of CBD on positive mood and good cognitive functions.

Study Design		CBD Dose *		Source		Route		Duration		Effects		Comment/Limitations		Reference
**Positive mood**														
Healthy volunteers (*n* = 17); randomized, double-blind, placebo-controlled, within-subject design		200, 400, 800 mg/day (2.9, 5.7, 11.4 mg/kg bw/day)		Synthetic CBD ([+]-CBD isomer) (liquid solution)		Oral		Acute; 4 sessions over 4 weeks, separated by at least 5 days		No consistent dose-dependent analgesia (CBD even increased pain on some measurements) Dose-dependent subtle effect on mood CBD produced small decreases in blood pressure		Acute administration Small sample size CBD isomer different to naturally occurring CBD CBD dose not adapted to subject’s weight Main research scope focused on CBD effects on pain (tested on healthy subjects). Technique used (cold pressor test) did not produce a reliable pain response. Contradictory results with respect to analgesia No specification if administration periods of CBD were homogenous among subjects. Subjective drug effects (including mood) based on VAS VAS results only shown for categories “stimulated”, “alert”, and “tired”, which could also be measurements for substance intoxication, and for drug ratings (“take again”, “good drug effect”, “bad drug effect”). No other VAS data for CBD subjective effects on mood available.		[56]
Overweight, healthy volunteers (*n* = 65); randomized, double-blind, placebo-controlled		15 mg/day (0.2 mg/kg bw/day)		Hemp-derived CBD (hemp oil extract in soft gel capsules)		Oral		Acute; 6 weeks		CBD was well tolerated (as judged by the general lack of side-effects and by hemodynamic and blood-based markers). Improvement (i.e., increase) of HDL cholesterol levels No changes in heart rate No overall significant changes on VAS measurements (including “mood in the past week”), but statistical tendencies for improvement of the “ability to cope with stress” and perceived life pleasure		Acute administration Single dose Overweight subjects CBD dose not adapted to subject’s weight HDL results contrast with other study that did not report significant differences in cholesterol levels (of note: CBD supplement used was different than the one used by Lopez et al.).		[155]
Healthy volunteers (*n* = 38); double-blind, placebo-controlled, within-subjects design		300, 600, 900 mg (300 mg/mL solution) (4.3, 8.6, 12.9 mg/kg bw/day)		Not specified (liquid solution)		Oral		Acute; 4 sessions, separated by at least 1 week		CBD was well tolerated (no adverse effects). No detectable subjective effects or alterations in mood or anxiety (ratings of momentary mood states) No effect on reactions to emotional stimuli on standardized tasks No anxiolytic effects At a dose of 900 mg, CBD slightly decreased attentional bias toward emotional facial expressions, and heart rate was slightly increased. No reduction of feelings of rejection		Acute administration CBD dose not adapted to subject’s weight Absence of pharmacokinetic data to reassure intended CBD plasma concentration No specification if administration periods of CBD were homogenous among subjects Conclusions based on subjective (mood) states/effects (POMS) Tests used (e.g., reaction time to detect a stimulus as a measurement of attentional bias) might not be sensitive enough. Mood effects of CBD might only be evident in individuals with high levels of anxiety.		[156]
**Good cognitive functions**														
Healthy volunteers (*n* = 17); randomized, double-blind, placebo-controlled, crossover design		15, 300, 1500 mg/day (0.2, 4.3, 21.4 mg/kg bw/day)		Synthetic CBD (100 mg/mL) in medium-chain triglyceride oil		Oral (administered together with a high-fat supplement)		Acute; four treatment sessions within 60 days (with a median washout period of 7.5 days)		No changes in cognitive function No changes on driving performance CBD reduced ratings in VAS related to “anxiousness”.		Acute administration Small sample size CBD dose not adapted to subject’s weight 12 participants had low but detectable levels of CBD due to residual levels from the previous administration. CBD administration together with high-fat supplement, which may increase CBD bioavailability Plasma CBD levels varied among participants		[162]
Healthy volunteers (*n* = 48); randomized, double-blind, placebo-controlled		50 mg/day (0.7 mg/kg bw/day)		Hemp-derived CDB		Oral		8 weeks		No improvement in aerobic or anaerobic fitness Prevention of reduction in peak anaerobic output No improvement in subjective and objective cognitive function (no differences in T-scores for cognitive function and cognitive abilities, including mental acuity, concentration, verbal and nonverbal memory, verbal fluency, and perceived changes in these cognitive functions) No improvement of psychological wellbeing aspects scores (including autonomy, self-acceptance, positive relation with others, personal growth)		Acute administration CBD dose not adapted to subject’s weight Surveys used to measure cognitive function might be insufficient to cover the diverse range of cognitive functions and abilities.		[90]

* Calculated for a person with 70 kg bw. CBD: cannabidiol; VAS: visual analogue scale; POMS: Profile of Mood States.

### 6.5. Anxiety

Anxiety is a disease state (ICD-10 code F41) [188]. Therefore, it is generally inappropriate to use anxiety-related claims in food information, which should not attribute any properties relating to the prevention, treatment, or cure of a human disease (e.g., Art. 7 of Regulation (EU) No 1169/2011). Health claims that food ingredients can “ameliorate subthreshold and mild anxiety” or that ingredients can “help to moderate signs of anxiety in mildly stress-sensitive adults” have been submitted by industry for other ingredients, but none of the anxiety claims are currently authorized according to the EU Register of Health Claims. Nevertheless, anxiety-related claims are often used in commercial information about CBD [12,189].

Research suggesting that CBD in *Cannabis* may counteract Δ^9^-THC -induced anxiety dates back to the 1980s [190,191], and numerous animal and human studies have been published since then. A review of animal studies (*n* = 17) in rats and mice found a general effect of CBD on anxiety [192], justifying further clinical research. Human studies investigating the impact of CBD on anxiety at doses ≤ 300 mg/day (4.3 mg/kg bw/day) are summarized in Table 6. Arnold et al. [193] recently reviewed the efficacy of CBD in humans considering the low-dose and high-dose ranges. In the low-dose range, three studies with a single administration of a CBD dose were included with anxiety as the primary outcome (100 mg/day or 1.4 mg/kg bw/day [194], 150 mg/day or 2.1 mg/kg bw/day [195], 150 mg/day or 2.1 mg/kg bw/day [196]). None of these trials showed effects of CBD in the dose range < 300 mg/day (4.3 mg/kg bw/day) on anxiety (Table 6). By contrast, all included studies investigating higher doses of 300 mg CBD/day (4.3 mg/kg bw/day) [72,106,194,195,197,198], 350 mg CBD/day (5 mg/kg bw/day) [199], or 400 mg CBD/day (5.7 mg/kg bw/day [57,74,200] reported positive effects on anxiety. However, some of these studies were conducted in patients with social anxiety disorders [198] or heroin use disorders [74], which may not be directly transferrable to healthy subjects, e.g., consumers of food supplements. Other studies, not included in the review by Arnold et al. [193], also showed positive effects at 300 mg CBD/day (4.3 mg/kg bw/day) [201], 600 mg CBD/day (8.6 mg/kg bw/day) ([115,202,203], and 800 mg CBD/day (11.4 mg/kg bw/day) [204], while other studies found no effects at 600 mg CBD/day (8.6 mg/kg bw/day) [112,195,205,206], 150–600 mg (2.1–8.6 mg/kg bw/day) [207], or even 900 mg CBD/day (13 mg/kg bw/day) [194]. Overall, there appears to be little support for a U-shaped dose–response as postulated in some studies [194,195], which are also limited by small study size combined with subjective measures. While preliminary data from a small open-label clinical trial (*n* = 14) seem to support the efficacy of a full-spectrum, high-CBD product for anxiety relief (35 mg CBD/day, corresponding to 0.5 mg/kg bw/day, 0.8 mg Δ^9^-THC), the effect may have been confounded by the relatively high concentration of Δ^9^-THC in the product. A double-blind, placebo-controlled phase of the trial is currently underway for definitive assessment [208]. Another double-blind, randomized placebo-controlled trial comparing 50 mg CBD (0.7 mg/kg bw/day) vs. 300 mg CBD (4.3 mg/kg bw/day) in 63 individuals for 2 weeks indicated no effects on worry severity or anxiety symptoms for the 50 mg dose, while 300 mg of CBD was reported to reduce anxiety symptoms [209]. Several other small studies, single case studies, and case series reported improvements in anxiety at lower doses (25–300 mg, 0.4–4.3 mg/kg bw/day) [210,211,212,213], and there are multiple other clinical trials on anxiety currently ongoing [214].

Of the various effects studied with CBD, anxiety relief at doses of 300–400 mg (4.3–5.7 mg/kg bw/day) has emerged as the most reproducible result [193]. In the various reviews, the interpretation of the evidence is highly inconsistent. On the one hand, evidence from human studies above 300 mg (4.3 mg/kg bw/day) has been described as being “strong” [215]. Similarly, CBD has been proposed as an effective treatment for anxiety-related disorders, as suggested by some of the strongest evidence currently available in the research field related to CBD research [216]. On the other hand, a systematic review of the dose–effect relationship of CBD in clinical and preclinical research came to more cautious conclusions. While anxiolytic effects tend to be reported in the 300–600 mg range (4.3–8.6 mg/kg bw/day), more data are needed to determine whether this range actually provides a reproducible anxiolytic effect. More investigations are needed to explain the loss of possible effects at higher concentrations observed in rodent studies. Indeed, the inconsistent linear effect of CBD in reducing anxiety calls into question its widespread use as an anxiolytic [217], especially since evidence between CBD and anxiolytic effects is reported to be of “low quality” [218] and research in this area is considered to be insufficient to support strong conclusions [219]. A systematic review that included an assessment of study quality using Cochrane criteria found that only 16% of the studies assessed were at “low risk” for all sources of bias, and the effectiveness of CBD was generally found to be unclear [220]. Peng et al. concluded that further research is needed to evaluate the efficacy of CBD, and to determine both the appropriate dose of CBD for the treatment of anxiety and the long-term safety of CBD use [30].

In conclusion, there is currently a lack of evidence linking CBD at doses up to 100–150 mg (1.4–2.1 mg/kg bw/day) with changes in anxiety. Therefore, anxiety-related claims such as “CBD helps to moderate signs of anxiety” are currently not validated for the dose range ≤ 300 mg/day (4.3 mg/kg bw/day) expected in foods. The same conclusion was reached by a study suggesting that over-the-counter (OTC) supplements (10–20 mg/day; 0.15–0.3 mg/kg bw/day) are unlikely to be effective because the lower doses studied had no effect on anxiety [221]. In agreement with Millar et al., larger, more robust clinical trials are needed to confirm the potential of lower oral doses of CBD to modulate anxiety [222].

### 6.6. Stress Management

In contrast to anxiety as a medical condition, stress is a natural human reaction to challenges and difficult situations that leads to a physiological response. However, perception of stress, and subsequent physiological effects, can considerably vary between individuals. Depending on the specific stress trigger and an individual’s personality, the stress response may be inappropriate or prolonged. Since the endocannabinoid system plays a crucial role in the modulation of stress responses and CBD is able to exert effects on the endocannabinoid system [223], CBD-containing foods/food supplements are advertised as stress management tools.

The effect of single, mostly high-CBD doses (up to 600 mg/day; 8.6 mg/kg bw/day) to reduce anxiety caused by extensive short-term stress has already been discussed in Section 6.5. The effects of CBD on stress symptoms in humans in the dose range ≤ 300 mg/day (4.3 mg/kg bw/day) are shown in Table 7. A single dose of 15 to 60 mg CBD (0.2–0.9 mg/kg bw/day) reduced Δ^9^-THC-induced stress responses such as mild anxiety and tachycardia [224]. In the study by Appiah-Kusi et al. (see Section 6.5), a dose of 600 mg CBD (8.6 mg/kg bw/day) not only reduced anxiety, but also the levels of the primary stress hormone cortisol [203]. The administration of CBD by itself increased sedation, changed heart rate variability, and lowered cortisol levels in saliva [225,226]. A further symptom of stress is fear, which is controlled via CB1 signaling. A dose of 32 mg CBD per day (0.5 mg/kg bw/day) removed the feeling of fear and aversive memory ([210]; see Section 6.5). Chronic stress can also lead to burnout syndrome, which is characterized by emotional exhaustion, anxiety, and depression. Several studies described improvement of burnout symptoms by CBD treatment at doses in the range of 300 to 330 mg (4.3–4.7 mg/kg bw/day) [72,201]. Patients with post-traumatic stress disorder (PTSD) caused by severe stress peaks are discussed as a target group for drug treatment with CBD (reviewed by [227]). Nevertheless, some studies showed insufficient evidence to manage PTSD by administering CBD (reviewed by [228,229]). In a retrospective case study on PTSD, 11 patients who received 25 to 100 mg CBD/day (0.4–1.4 mg/kg bw/day) in oil or as capsules over an 8-week period showed a decrease in PTSD symptom severity. However, the patients received concurrent psychiatric treatment, including psychiatric medications and psychotherapy [230]. An upcoming clinical trial, investigating the effects of CBD doses of 300 mg per day (4.3 mg/kg bw/day) in patients with PTSD will help to clarify whether CBD may be an effective treatment for stress disorders [231].

Overall, there are no scientific data to support the use of CBD as a medication for stress. Some studies showed positive effects of CBD on burnout syndrome or PTDS. However, most studies assessed CBD effects at doses within the therapeutic range. Additionally, subjects receiving CBD were frequently undergoing concomitant medical interventions.

### 6.7. Relaxation

The term “relaxation”, when used as such, is typically used to qualify a state of emotional and mental well-being, rather than the relaxation of a specific organ system (e.g., muscle relaxation or vasodilation; for these effects see Section 6.1). Relaxation has also been defined as an activity that reduces the feelings of tension and the effects of stress [233]. In the context of food supplements, relaxation may refer to the ability of a supplement to help relieve feelings of anxiety, stress, and tension, and instead to feel calm and at ease. Relaxation can manifest in both physical sensations, such as a decrease in muscle tension, and psychological changes, such as a decrease in racing thoughts or worry. It can also lead to an improved sense of well-being and a general feeling of being at ease.

The claim “relaxation” is on the borderline between being a non-specific health claim (which needs to be accompanied by a specific health claim) and being a specific health claim by itself. EFSA has evaluated the term “relaxation” as a specific health claim for several substances, including caffeine and melatonin [234,235]. However, a “relaxation” claim is currently not permitted because EFSA could not conclude that a cause-and-effect relationship had been established between the consumption of the respective foods and relaxation.

Relaxation is a concept commonly associated with psychotropic cannabinoids such as Δ^8^- and Δ^9^-THC [236,237,238] and is one of the main reasons why people use Cannabis [239]. Similarly, for CBD, there is ample evidence that consumers associate its use as a lifestyle supplement with “relaxation”. For example, CBD use for relaxation is promoted online, on social media, and by influencers [14,240], and it is one of the most common marketing claims for CBD [9].

Since relaxation is such a vague concept and thus not sufficiently defined for a scientific evaluation, the literature review did not reveal a single study on CBD that examined the endpoint “relaxation”. Therefore, there is currently no evidence that CBD (at any dose) contributes to or maintains relaxation beyond placebo effects.

### 6.8. Sleep

Sleep disturbances are often cited as an indication for treatment with medicinal Cannabis. There is an increasing clinical interest in the therapeutic potential of cannabinoids in the treatment of sleep disorders. Mechanistically, there is evidence that the endocannabinoid system is involved in the regulation of the circadian sleep–wake cycle [241]. There is a variety of studies that investigated the effects of plant-derived cannabinoids, such as Δ^9^-THC, but also CBD on sleep disorders such as insomnia and sleep apnea. Some limitations of most of these studies are the reduced small sample size and the lack of control groups. Also, many studies investigated sleep only as a secondary outcome. Whereas a variety of reviews concluded that there is some evidence that Δ^9^-THC may have an effect, there is no convincing data available demonstrating effects of CBD in the treatment of sleeping disorders. Clinical trials with CBD and Δ^9^-THC combinations [242,243] demonstrated some evidence that this combination may result in an improvement of subjective sleep parameters. However, single CBD applications may result in activation rather than in a mild sleep promoting effect. Systematic reviews on this topic came to different conclusions. In a systematic review analyzing 31 studies, improvements of sleep quality could be detected in 7 out of 19 randomized and 7 out of 12 uncontrolled trials in patients with pain release disorders [244]. No effects were detected in healthy patients. The authors concluded that high-quality evidence to support Cannabis use for sleep disorders remains limited. In another systematic review focusing on CBD, the authors analyzed 43 studies using CBD or CBD/Δ^9^-THC combinations. Four of seven studies using single CBD therapy reported a significant improvement in insomnia outcomes. These authors concluded that CBD alone may be beneficial to alleviate symptoms of insomnia [245].

Human studies addressing the effect of CBD on sleep disorders at doses ≤ 300 mg/day (4.3 mg/kg bw/day) are summarized in Table 8. In a crossover, double-blind design study, Linares et al. evaluated how CBD administered at a clinically anxiolytic dose affected the sleep–wake cycle of healthy subjects [246]. In a double-blind randomized design study, twenty-seven healthy volunteers were assigned to receive either 300 mg of CBD or a placebo on the first night. The following night, the same protocol was carried out with the substance that had not been used on the first occasion. CBD or a placebo were administered 30 min prior to the start of the 8 h polysomnography recordings. Right after polysomnography, cognitive and subjective measurements were conducted to evaluate possible residual effects of CBD. There were no significant effects of CBD (*p* > 0.05). The authors concluded that acute CBD administration at an anxiolytic dose does not seem to interfere with the sleep cycle of healthy subjects.

Bonn-Miller et al. assessed the effects of CBD and cannabinol (CBN) in different combinations in a double-blind, randomized, placebo-controlled study [247]. Participants were randomized to receive either (a) placebo, (b) 20 mg CBN, (c) 20 mg CBN + 10 mg CBD (0.14 mg CBD/kg bw/day), (d) 20 mg CBN + 20 mg CBD (0.3 mg CBD/kg bw/day), or (e) 20 mg CBN + 100 mg CBD (1.4 mg CBD/kg bw/day) for seven consecutive nights. The following endpoints were assessed: sleep quality (primary endpoint), sleep onset latency, number of awakenings, wake after sleep onset (WASO), general sleep disturbance, and daytime fatigue (secondary endpoints). Participants receiving 20 mg CBN showed fewer night-time awakenings and less overall sleep disturbance compared to the placebo group, with no effect on daytime fatigue. The effects of CBN were not enhanced by the addition of CBD.

Kisiolek et al. studied the effects on mental health, sleep quantity and quality, and immune cell function in healthy, college-aged subjects after administration of 50 mg oral CBD (0.7 mg CBD/kg bw/day) during 8 weeks [248]. Twenty-eight participants (mean age: 25.9 ± 6.1 years old) were randomly assigned to take either daily oral capsules containing 50 mg of CBD or a calorie-matched placebo. Anthropometric measurements, mental health questionaries, sleep evaluations, and immune function were assessed before and after CBD administration. In this study, the CBD group showed significant improvements in sleep quality, assessed through a sleep questionnaire (*p* = 0.0023).

Saleska et al. investigated combinatory effects with melatonin on sleep quality [249]. Participants (*n* = 1793 adults experiencing symptoms of sleep disturbance) were randomly assigned to six groups to receive a combination of 15 mg CBD/day (0.2 mg/kg bw/day) with other cannabinoids and/or melatonin for 4 weeks: 15 mg CBD + 15 mg CBN + 5 mg melatonin, 15 mg CBD + 15 mg CBN, 15 mg CBD alone, 5 mg melatonin alone, 15 mg CBD + 15 mg CBN, or 15 mg CBD +15 mg CBN + 5 mg cannabichromene (CBC) [249]. Sleep disturbance was assessed weekly over a period of 5 weeks (baseline week and 4 weeks of product use) by using the Patient-Reported Outcomes Measurement Information System (PROMIS™) Sleep Disturbance SF 8A. Most participants (56% to 75%) reported a clinically relevant improvement in their sleep quality across all dosing scenarios. The authors concluded that chronic use of CBD at low doses is safe and has the potential to improve sleep quality. However, these effects did not exceed those of 5 mg melatonin. Moreover, the concomitant administration of low doses of CBN and CBC may not improve the effects of formulations containing CBD or melatonin isolate.

Narayan et al. assessed the efficacy of 150 mg of CBD (2.1 mg/kg bw/day) as a sleep aid for primary insomnia in a randomized, placebo-controlled parallel design study, with a single-blind placebo run-in week followed by a two-week double-blind randomized dosing period [250]. Participants (*n* = 15 CBD product; *n* = 15 placebo) consumed the designated treatment sublingually one hour prior to bedtime. Daily sleep was assessed by wrist-actigraphy and sleep diaries. During four in-lab visits, sleep quality, sleep effort, and overall well-being were assessed. Regarding most sleep outcomes, supplementation of 150 mg CBD was comparable to placebo, whilst improving well-being, suggesting more prominent psychological effects.

The number of studies addressing the issue of sleep quality in healthy individuals is still limited, and the outcomes of the respective studies are conflicting. Therefore, there is currently a lack of evidence suggesting that CBD (at any dose) helps to improve sleep quality.

### 6.9. Pain

Recent surveys indicate that 62% of CBD consumers intend to treat a disease state, of which chronic pain, anxiety, and arthritis are the most frequent [251]. However, even though pain is one of the health claims most frequently addressed in relation to CBD use, most data available are from animal studies [252].

The effects of CBD on chronic pain in humans at doses ≤ 300 mg/day (4.3 mg/kg bw/day) are summarized in Table 9. Frane et al. evaluated the efficacy of CBD for the treatment of arthritis by a novel anonymous questionnaire [253]. A self-selected convenience sample (*n* = 428) was recruited between 5 May 2020 and 5 November 2020. To ascertain variations in arthritis types and enhancement in quality-of-life symptoms, statistical analysis was conducted. In addition, variables associated with reducing or disrupting other medications were identified by a regression analysis. Using CBD was linked to improvements in pain (83%), sleep quality (66%), and physical function (66%). Analysis of diagnostic subgroups (osteoarthritis, rheumatoid, or other autoimmune arthritis) noted improvements in physical function between the groups (*p* = 0.013), especially in the osteoarthritis group. After using CBD, the whole cohort reported 44% less in pain (*p* < 0.001). The group with osteoarthritis experienced a higher percentage (*p* = 0.020) and point (*p* < 0.001) reduction in pain than those with rheumatoid or other autoimmune arthritis. The majority of respondents stated that they reduced or discontinued other medications after the use of CBD.

Capano et al. assessed the effect of CBD (full hemp extract formulation) on indicators for quality of life and opioid use among patients with chronic pain [254]. As an initial sample, 131 patients from a private pain management center were asked to participate, 97 of whom completed the 8-week study. Patients between 30 and 65 years of age with chronic pain who had taken opioids for at least 1 year were included. Data were collected at the beginning of the study and after 4 and 8 weeks. The participants received a bottle with sixty soft gels each containing 15 mg of hemp-derived CBD. Almost all participants (91) took two soft gels daily (~30 mg CBD, equivalent to ~0.4 mg/kg bw/day). After including CBD-rich hemp extract to their treatment, 53% of the chronic pain patients reduced or stopped using opioids within 8 weeks, and 94% of CBD users reported an improvement in life quality.

Vela et al. investigated CBD as an additional pain-relieving therapy in patients with hand osteoarthritis or psoriatic arthritis who have moderately severe pain despite therapy [255]. According to a randomized, double-blind, placebo-controlled study design, patients received 20 to 30 mg (0.3–0.4 mg/kg bw/day) synthetic CBD or placebo daily for 12 weeks. The primary outcome measured was pain intensity during the previous 24 h. A total of 129 patients were included in the primary analysis. No clinically or statistically significant effects of CBD on pain intensity were found in patients with psoriatic arthritis or hand osteoarthritis compared with placebo.

Zubcevic et al. assessed the effect of Δ^9^-THC, CBD and their combination on peripheral neuropathic pain [256]. A total of 145 patients were included in a randomized, double-blind study with four treatment arms, namely placebo, CBD, Δ^9^-THC, and the combination of CBD and Δ^9^-THC (CBD/Δ^9^-THC). They were treated for 8 weeks with flexible drug doses (CBD (5–50 mg; 0.07–0.7 mg/kg bw/day), Δ^9^-THC (2.5–25 mg), and CBD/Δ^9^-THC (5 mg/2.5 mg–50 mg/25 mg)). Compared to placebo, none of the treatments reduced pain (*p* = 0.04–0.60).

De Vita et al. investigated the effects of a single administration of 50 mg CBD (0.7 mg/kg bw/day) and the expectancies of pain relief on experimental pain reactivity in healthy adults [257]. In a study with crossover, 2 × 2 factorial balanced placebo design, drug administration (given active CBD or inactive substance) and verbal orders (subjects were informed whether they were receiving active CBD or inactive substance) were experimentally manipulated. The main focus was on pain threshold, tolerance, intensity, unpleasantness, conditioned pain modulation (CPM), and offset analgesia (OA). Post-manipulation pain assessments were performed after a 30 min absorption period. In four experimental sessions, the test subjects were randomly exposed to the different manipulation conditions: control (told inactive and given inactive); expectancy (told active but given inactive); drug (told inactive but given active); and expectancy + drug (told active and given active). Authors found that CBD-induced reductions in pain unpleasantness were achieved both through pharmacological administration of CBD and psychological expectancies for receiving a CBD analgesic.

Arout et al. investigated the analgesic effects of CBD of a range of oral CBD doses in healthy humans [56]. In a double-blind, placebo-controlled trial, doses of 200, 400, and 800 mg CBD (2.9, 5.7, and 11.4 mg/kg bw/day) were given orally to healthy non-cannabis-using volunteers (*n* = 17; 8 men, 9 women). Pain sensitivity was tested according to a cold pressor test. CBD failed to consistently affect the pain threshold and tolerance relative to placebo.

Bebee et al. assessed the analgesic efficacy and safety of a single oral dose of CBD as an adjunct to standard care for patients with acute low back pain [258]. One hundred patients were randomized to receiving 400 mg CBD (5.7 mg/kg bw/day) or placebo in addition to standard analgesic medication. The pain score was assessed two hours after CBD or placebo administration. In this study, CBD was not superior to placebo. 

In a randomized, double-blind, placebo-controlled, repeated-dose pilot study, Peters et al. investigated the effects of CBD and CBG on the recovery from DOMS [96]. The formulation tested had no discernible impact on objective recovery metrics but resulted in a moderate decrease of self-reported average soreness/discomfort 72 h post-DOMS, although the authors stated that these preliminary observations should be interpreted with caution (see also Section 6.1).

Cochrane et al. investigated the daily intake of 150 mg/day CBD (2.1 mg/kg bw/day) for 3 days (divided into two doses of 75 mg each) during the recovery process after eccentric training [92]. In a double-blind, crossover study, 13 untrained men completed 6 sets of 10 isokinetic-eccentric biceps curls. Muscle soreness was analyzed. No effects of CBD could be determined.

Van Orten-Luiten et al. examined the effect of a chewing gum containing 50 mg CBD (~0.7 mg/kg bw/day) on abdominal pain and perceived well-being in a double-blinded, randomized, placebo-controlled trial with crossover design [232]. Chewing gums were taken according to need and severity of the pain, with a maximum of six pieces per day (300 mg CBD/day or ~4.3 mg/kg bw/day). At a group level, the results showed no statistically significant difference in pain scores between placebo and CBD.

In a single-arm study [259], young girls (*n* = 12; age range: 12–24 years old) suffering from chronic pain and somatoform psychosis due to the vaccination against human papillomavirus vaccine were examined for short-term effects of CBD-enriched hemp oil, administered sublingually to relieve symptoms and improve quality of life. The girls’ pain issues included chronic headache, gastrointestinal pain, fibromyalgia, arthralgia, and myalgia. The starting dose was 25 mg CBD per day (0.4 mg/kg bw/day), which was gradually enhanced to a maximum dose of 150 mg of CBD per day (2.1 mg/kg bw/day) at week 6–7 and then gradually reduced back to the initial dose. For eight patients (66.7%), data could be generated; four patients stopped treatment due to adverse events or lack of improvement. Compared to baseline, patients who received the CBD-enriched hemp oil exhibited no significant reduction in body pain, vitality, physical component score, and perceived functioning in the social role, according to the SF-36 questionnaire (36-Item Short-Form Survey Instrument: validated questionnaire used to measure self-reported quality-of-life measures) (*p* < 0.05 for each).

A second open-label, single-arm trial assessed CBD for pain relief in kidney transplant patients. Patients (*n* = 7; age range: 58–75 years old) who had undergone a renal transplant and were asking for pain relief received increasing oral doses of 50 to 150 mg CBD twice a day (1.4–4.3 mg/kg bw/day) for 3 weeks [260]. Within the first 15 days, two patients experienced complete pain improvement, four experienced a partial response, and one had no change at all. Statistical tests were not used. It cannot be differentiated whether the benefits seen in the study were due to CBD, placebo effect, or natural reduction of symptoms.

Wade et al. investigated pain and spasticity in a randomized, double-blind, multigroup crossover trial [261]. Patients (*n* = 24) with spinal cord injury, brachial plexus damage, limb amputation due to neurofibromatosis, or multiple sclerosis were included in this study [261]. They received 2.5–120 mg CBD (0.04–1.7 mg/kg bw/day), Δ^9^-THC, CBD + Δ9-THC, or placebo for 2 weeks during each phase of the trial in a crossover fashion. Pain, coordination, spasm, spasticity, and bladder function were determined daily according to a self-administered visual analogue scale (VAS) (0 = worst to 100 = best) over the last 7 days of each phase, and averaged. Compared to the placebo group, pain control was significantly better in the CBD group.

In conclusion, based on animal studies and a very limited number of human intervention studies and cross stationary investigations, there is some evidence that CBD may be effective for the treatment of chronic pain in adults. However, the evidence remains limited since the beneficial effects attributed to the use of CBD have often been inconsistent across different studies. Based on the limited data available, a specific conclusion regarding CBD and arthritis pain, joint pain, or arthrosis pain is not possible.

**Table 9:** The impact of CBD on pain.

Study Design		CBD Dose *		Source		Route		Duration		Effects		Comment/Limitations		Reference
Human														
Girls with significant somatoform psychological and chronic pain as a result of the human papillomavirus vaccine, 12–24 years old (*n* = 12); single-arm trial		25 mg/day increasing to 150 mg/day (0.4–2.1 mg/kg bw/day)		CBD-enriched hemp oil		Oral		12 weeks		SF-36 pain questionnaire showed significant benefits in the physical component score (*p* < 0.02), vitality (*p* < 0.03), and social role functioning (*p* < 0.02) after the treatment. The administration of hemp oil also significantly reduced body pain according to the SF-36 assessment.		Limited number of participants No placebo control Not blind design Dose changing over the 12-week treatment period		[259]
Patients with a mean age of 64.5 years old (range, 58–75 years old), (*n* = 7); open-label, single-arm trial		Increasing doses of oral CBD (50 to 150 mg twice a day;(1.4–4.3 mg/kg bw/day)		CBD hemp oil		Oral		3 weeks		Two patients had total pain improvement, four had a partial response in the first 15 days, and in one there was no change.		Limited number of participants No placebo control Not blind design Dose changing over the 3-week treatment period		[260]
Patients with multiple sclerosis, spinal cord injury, brachial plexus damage, and limb amputation due to neurofibromatosis (*n* = 24); randomized, double-blind, multigroup crossover design		2.5–120 mg/24 h (0.04–1.7 mg/kg bw/day)		Whole-plant extracts of Δ^9^-THC (THC-rich CME), cannabidiol (CBD-rich CME) and a 1:1 preparation of the two (THC:CBD).		Sublingual spray		2 weeks		Pain relief associated with both THC and CBD was significantly superior to placebo.		Dose was chosen by participants		[261]
131 cancer patients taking opioids		30 mg/day (0.4 mg/kg bw/day)		Hemp-derived CBD soft gels		Oral		8 weeks		Approx. half of chronic pain patients (53%) reduced or eliminated their opioids within 8 weeks after adding CBD-rich hemp extract to their regimens. Almost all CBD users (94%) reported quality of life improvements.		Not blind design Activity together with opioids		[254]
Patients with hand osteoarthritis or psoriatic arthritis experiencing moderate pain intensity despite therapy (*n* = 129); randomized, double-blind, placebo-controlled		20–30 mg/day (0.3–0.4 mg/kg bw/day)		Synthetic CBD		Oral		12 weeks		Neither clinically nor statistically significant effects of CBD for pain intensity in patients with hand osteoarthritis and psoriatic arthritis were detected when compared with placebo.				[255]
Patients with painful polyneuropathy (*n* = 145); randomized, double-blind		5–50 mg/day (0.07–0.7 mg/kg bw/day)		Not clear		Oral		8 weeks		None of the treatments reduced pain compared to placebo (*p* = 0.04–0.60).				[256]
Healthy men and women (18–30 years old); double-blind, crossover, balanced placebo 2 × 2 factorial design		50 mg/day (0.7 mg/kg bw/day)		Hemp-derived CBD		Oral		Single administration		Authors found that cannabinoid-induced reductions in pain unpleasantness were caused by both psychological expectancies for receiving a CBD analgesic and pharmacological administration of CBD.		Single administration Psychological design		[257]
Healthy men and women (32 ± 8 years old) (*n* = 17); double-blind, placebo-controlled, crossover design		200, 400 g, 800 mg/day (2.9, 5.7, 11.4 mg/kg bw/day)		Pure CBD		Oral		Single treatment		CBD failed to consistently affect pain threshold and tolerance in the CPT relative to placebo. CBD had dose-dependent, modest effects on mood and subjective drug effect.		Single administration		[56]
Male and female patients (34–60 years old) with acute, non-traumatic low back pain (*n* = 100); randomized, double- blinded, placebo-controlled clinical trial		400 mg/day (5.7 mg/kg bw/day)		Synthetic CBD 99.9% purity		Oral		Single treatment		CBD was not superior to placebos.		Single administration		[258]
Healthy males (18–65 years old) (*n* = 40; *n* = 20 per group); randomized, double-blind, placebo-controlled, repeated-dose pilot study		70 mg CBD (1 mg/kg bw/day) and 100 mg CBG per day		Water-soluble liquid. CBD oil nano-particularized, and then combined with other ingredients to maintain the suspension.		Oral		3.5 days		Modest effects on decreased self-reported average soreness/discomfort 72 h post-DOMS		CBD was given in combination with CBG. Effects only on markers that are self-reported		[96]
Untrained men (21.85 ± 2.73 years old) (*n* = 13); double-blind, placebo- controlled, crossover design		150 mg/day (2.14 mg/kg bw/day)		CBD oil		Oral		3 days		No effect on muscle soreness could be detected.		Only non-invasive markers have been determined		[92]
Female patients with irritable bowel syndrome, 22–50 years old (*n* = 32); randomized, double-blinded, placebo- controlled crossover design		50–300 mg/day (0.7–4.3 mg/kg bw/day)		CBD-containing chewing gum		Oral		8 weeks		There was no statistically significant difference in pain scores between CBD and placebo at a group level. Subgroup and individual analyses showed a highly variable picture.		Markers for the direct response of the immune system to CBD were not investigated in this study.		[232]

* Calculated for a person with 70 kg bw. CBD: cannabidiol; DOMS: delayed onset muscle soreness; Δ^9^-THC: delta-9-tetrahydrocannabinol.

### 6.10. Menstrual Discomfort

Menstrual-related symptoms (MRS) include pain (uterine pain, abdominal cramps, and back pain, together referred to as chronic pelvic pain (CPP) or primary dysmenorrhea (PDM)), migraines, anxiety, and sleep disorders, as well as digestive symptoms such as diarrhea, constipation, nausea, flatulence, and vomiting. The terms premenstrual syndrome (PMS) or premenstrual dysphoric disorder (PMDD) are often used to describe severe symptoms before menstruation. Regardless of the diagnosed conditions, CBD is advertised as a natural remedy for pain, anxiety, and sleep disorders (see also Section 6.5, Section 6.8 and Section 6.9) and is also marketed with health claims related to MRS and CPP.

#### 6.10.1. Studies on CBD and Menstrual Discomfort

There is only one dissertation-style study that examined isolated CBD as a treatment for menstrual-related pain, depression, anxiety, and sleep disorder [262]. Thirty-three women who suffered from moderate to severe MRS received 160 mg or 320 mg CBD daily (2.3–4.6 mg/kg bw/day) for five consecutive days each month, beginning on the first day that MRS symptoms appeared. The effects on patients’ well-being were assessed with professionally validated standard questionnaires. Slight improvements in MRS were noted on these self-report measures. The author concluded that further studies are needed to confirm the effects of CBD on menstrual discomfort [262].

An upcoming clinical trial (drug development phase II) will evaluate the efficacy of CBD alone (200 mg/day; 2.9 mg/kg bw/day) compared to the conventional use of the analgesic ibuprofen for the relief of menstrual pain [263]. CBD (in a range between 10 and 200 mg/day (0.14–2.9 mg/kg bw/day)) was also tested in combination with Δ^9^-THC, analgesics or hemp oil as a remedy for menstrual cramps, but these studies do not allow a dose–response derivation for CBD alone [259,264] (https://classic.clinicaltrials.gov/ct2/show/NCT04527003, accessed on 19 November 2024). There are no further studies on CBD as a remedy for menstrual discomfort, MRS, or CPP.

#### 6.10.2. Therapy of Endometriosis

Endometriosis is considered one of the causes of secondary dysmenorrhea (SDM), in which ectopic endometrial cells exhibit abnormal proliferation and apoptosis regulation in response to certain stimuli and cause severe abdominal pain. In endometriosis, several signaling pathways have been studied that correlate with abnormal proliferation or growth of endometrial cells. The female reproductive system has a high number of cannabinoid receptors that affect angiogenesis, proliferation, and fibrosis, and endocannabinoids are involved in the regulation of the signaling pathways responsible for the development of endometriosis [265,266,267]. Due to its involvement in the development of endometriosis and associated pain, the cannabinoid receptor CB1 was proposed as a potential treatment target of CBD [268,269,270,271].

A study in endometriosis-induced mice suggested that the antifibrotic, antioxidant, and anti-inflammatory activities of CBD may be beneficial in counteracting the development of endometriosis and the resulting chronic pain. It was shown that CBD at higher doses within the therapeutic dose range (10 mg/kg bw/day) led to histological changes in endometriosis tissue and influenced inflammatory and oxidative processes [272]. There are no further studies of CBD or other cannabinoids on symptoms of endometriosis. Of note, the UK national guidance does not recommend Cannabis-based products or CBD to patients with endometriosis and chronic pelvic pain, owing to the lack of clear evidence of benefits, and demanded more comprehensive research into the impact of CBD/endocannabinoids in the context of endometriosis [273].

In conclusion, there are almost no data on the effect of CBD on MRS, and especially on associated CPP. In the few planned clinical trials, doses between 60 and 200 mg CBD/person (0.9–2.9 mg/kg bw/day) will be investigated, but sometimes only in combination with other substances. Data available so far do not support the use of CBD for the relief of menstrual pain. Similarly, the few data available on effects of CBD on endometriosis do not provide clear evidence for beneficial effects of CBD, particularly in the dose range relevant for foods/food supplements.

## 7. Exposure Considerations

Due to uncertainties and data limitations, particularly with regard to consumer habits, reliable estimation of CBD exposure from food supplements is not possible. Manufacturers of CBD oils often do not provide dose recommendations, and labeling of CBD products is often misleading or even wrong. A recent study showed that CBD concentrations of commercial CBD oils were on average 21% higher than stated by the manufacturer, which could lead to a higher intake than intended by consumers [4]. Multiple “CBD dose calculators” and dosage recommendations for various indications can be found online (some examples can be found in Appendix A, Table A1, or [274]). However, these dose calculators lack scientific support and estimate doses of approx. 1 to 1500 mg CBD per day (0.014–21 mg/kg bw/day) depending on the user’s input.

Dose recommendations found on the Internet are frequently based on a publication by Leinow and Birnbaum [275] and typically suggest micro, standard, and macro dosage, depending on the indication, severity of the condition, and body weight. For first-time CBD users and for treatment of mild conditions such as stress or sleep disorders, a dose of 0.5–30 mg CBD/day (0.007–0.4 mg/kg bw/day) is frequently recommended. Standard dosages for the treatment of, e.g., depression, arthritis, and moderate pain typically range from 10–115 mg CBD/day (0.14–1.6 mg/kg bw/day), while macro dosages of 50–1500 mg CBD/day (0.7–21 mg/kg bw/day) are recommended for severe conditions such as epilepsy, severe pain, and psychological problems (Table 10). The number of drops of CBD oil corresponding to these different dose ranges depending on the CBD content of the oil (5, 10, 20, or 40% CBD) are shown in Table 10. Assuming that one drop of CBD oil is about 0.033–0.05 mL (33–50 µL), one drop of CBD oil (50 µL) can contain, dependent on the CBD content, up to 2.5–20 mg CBD. To reach the recommended CBD lowest starting dose of 0.5 mg CBD/day, less than one drop of CBD oil regardless of its CBD content is needed. More importantly, the therapeutic starting dose of Epidyolex^®^ of 300 mg/day is readily achieved via consumption of 15 drops of a 40% CBD oil.

## 8. Risk Characterization

Currently, a wide range of CBD food products is available on the market. However, none of these products have been authorized in the EU as novel foods. CBD is only approved as a drug, Epidyolex^®^, as an adjuvant treatment of rare forms of early childhood epilepsy and tuberous sclerosis complex. The therapeutic starting dose of Epidyolex^®^ is 5 mg/kg bw/day (300–350 mg/day for a person of 60–70 kg, split into two daily doses), which is the lowest dose tested in humans in the Epidyolex^®^ authorization dossier (as well as in other human studies). At this dose, side effects on the liver (e.g., alteration of liver enzyme levels, increased liver enzyme activity in the blood), the central nervous system (e.g., somnolence, sedation), and the gastrointestinal tract (e.g., diarrhea) as well as drug interactions have been reported. As no NOAEL could be identified from these studies, the highest dose of CBD that does not cause adverse effects remains to be established. Effects on the liver have also been reported in monkeys, dogs, rats and mice. There is also evidence of adverse effects of CBD on male fertility as well as on the thyroid and adrenal glands in laboratory animals.

In its statement on the safety of CBD as a novel food, EFSA concluded that the safety of CBD as a novel food cannot be established at present, given the significant uncertainties and data gaps [2]. In 2021, the Swiss Federal Food Safety and Veterinary Office (FSVO) assessed the potential health risks associated with the consumption of food supplements containing CBD. Given the poor quality of most studies available at that time, no reliable conclusions with respect to the safety of CBD could be drawn. The FSVO noted that, until a comprehensive risk assessment is possible, a quantitative assessment should be conducted at least for liver toxicity in humans [25]. According to the FSVO, increased liver enzyme activities were observed in the blood in all human studies conducted in connection with the approval process of the medicinal product Epidyolex^®^. In order to estimate the risk, the study in healthy adults without concomitant medication in which 5 mg CBD/kg bw/day was administered orally for three weeks [73] was used. Significantly increased liver enzyme activities were observed in the blood compared to the control group. Applying an uncertainty factor of 30 (10 for intra-human variability, 3 for the consideration that effects occurred already at the lowest dose tested and therefore no NOAEL is available), the FSVO estimated an oral daily dose of 12 mg CBD/adult (0.17 mg/kg bw/day for a 70 kg person), which should not be exceeded. It was noted that, depending on the CBD content and the amount of hemp products ingested, this dose may be exceeded. According to the FSVO, long-term CBD consumption and the simultaneous intake of medication should be avoided, particularly given the potential for drug interactions [25].

The US FDA also strongly advises against the use of CBD due to the risk of liver injury, negative effects on male reproduction, and drug interactions, among other risks, especially during pregnancy or while breastfeeding [276,277].

Lachenmeier et al. used the point of departures (PODs) from animal and human data to propose HBGVs [278]. The authors suggested using a benchmark dose lower one-sided confidence limit for 10% extra risk (BMDL_10_) of 20 mg CBD/kg bw/day as a POD for the occurrence of centrilobular hypertrophy in the liver. This was derived from a 26-week oral study in rats (0, 15, 50, and 150 mg CBD/kg bw/day) [67] and is the lowest, i.e., most conservative, value from the available animal studies. The application of a default uncertainty factor of 100 (10-fold for interspecies differences and 10-fold for interindividual differences) results in a HBGV of 0.20 mg/kg body weight/day (14 mg/day). For the derivation of a HBGV based on human data, the authors proposed using the LOAEL of 4.3 mg/kg bw/day CBD from Crippa et al. [72] and applying an uncertainty factor of 30 (10 for intra-human variability and 3 for extrapolation from LOAEL to NOAEL), which would lead to an HBGV of 0.14 mg/kg bw/day or 10 mg/day. The authors suggested using the human HBGV of 0.14 mg/kg bw/day for risk assessment, but also pointed out that the HBGV values derived from animal and human studies are in very good agreement and that the animal data support the results in humans [278].

In the UK, new unpublished proprietary data from the industry were submitted and re-evaluated in the context of novel food applications. Based on this new evidence, in 2023, the UK Advisory Committee on Novel Foods and Processes (ACNFP) and the Committee on Toxicity (COT) established a provisional ADI of 0.15 mg/kg bw/day or 10 mg of 98% pure CBD per day for an average 70 kg adult as an ingredient in food [66].

The updated advice of the ACNFP/COT Committees was based on PODs from three pivotal 90-day repeated dose toxicity studies in rats with different CBD extracts (≥98% purity) provided by the industry (unpublished propriety data). These allow the identification of NOAELs for effects on the liver in rodents that were considered relevant to humans [66]. The PODs were divided by the default uncertainty factor of 100, and an additional uncertainty factor of 3 was applied to account for the sub-chronic study design. The PODs identified from the three studies were 72, 50, and 25 mg/kg bw/day and would lead, after applying an overall uncertainty factor of 300, to ADIs of 0.24, 0.17, and 0.08 mg/kg bw/day, respectively [66]. The committees agreed that there were observable adverse effects linked to the administration of CBD (Epidyolex^®^ formulation) to humans [66]. These effects were most notably in the form of hepatic injury at a CBD dose of ≥5 mg/kg bw/day (approx. 350 mg/person), inhibitory interactions with certain medications at a CBD dose of ≥1 mg/kg bw/day (approx. 70 mg/person), and somnolence at ≥10 mg/kg bw/day (approx. 700 mg/person) [26,27,66]. Furthermore, it was noted that reproductive toxicity and effects on the development of the offspring were observed in animal studies [26,27,66]. Given the physicochemical properties of CBD, it was considered likely that CBD is transferred into breastmilk, which could consequently pose a risk to nursing infants [26,27,66].

The ACNFP/COT established a provisional ADI of 10 mg/day for a 70 kg healthy adult based on the available data in animals and humans (calculated as the average of the ADIs based on the three pivotal studies (0.16 mg/kg bw × 70 kg), rounded to one significant figure) [66]. For the derivation of this provisional ADI, available human data, such as the existing evidence of drug–drug interactions at 1 mg/kg bw/day (70 mg/day) in humans, has been taken into account [66].

Thus, based on this new assessment, the UK FSA has issued new precautionary advice on CBD, recommending healthy adults should limit their consumption of CBD from food to 10 mg per day, e.g., 4–5 drops of 5% CBD oil (98% purity) [24]. Although the ACNFP/COT recommendation refers to a subset of CBD products, the FSA has issued a recommendation for all CBD products as a precautionary measure to provide clear communication to the public. It was stated that there is no acute safety risk associated with the consumption of more than 10 mg of CBD a day, based on the data currently available [24]. However, it was also noted that consumption above this level and over a period of time has been associated with some adverse effects on the liver and thyroid [24]. In 2024, the provisional ADI of 10 mg/day for a healthy 70 kg adult was retained as applicable to novel foods containing synthetic CBD of >98% purity [279].

Another potential risk of CBD oils is that they often also contain Δ^9^-THC, as evidenced by a recent study in which most of the samples analyzed contained Δ^9^-THC in quantities ranging from 5 to 1576 mg per kilogram (mean = 536 mg Δ^9^-THC/kg [3,4,5,6]). If such CBD oils are consumed, the acute reference dose (ARfD) for Δ^9^-THC of 1 μg/kg bw, established by the EFSA [7], may be exceeded. Indeed, Lindekamp et al. demonstrated that in 38% of the CBD oil samples (*n* = 26), just 2 to 10 drops, which is a common consumption recommendation by manufacturers for adults, would be sufficient to exceed the ARfD [4].

**Figure 2:**
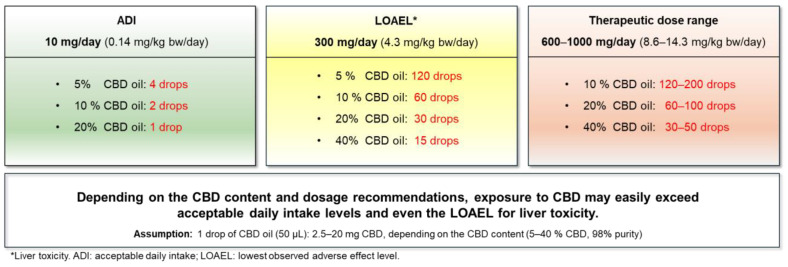
Exposure considerations for the consumption of CBD oils on the market.

According to EFSA’s guidance on the risk–benefit assessment of foods, conservative estimates of the human dietary exposure are compared with the HBGV in a Tier 1 assessment [28] (see also Section 2). The most recent UK assessment (2023) [24] and the LOAEL of 300 mg CBD/day equivalent to 4.3–5 mg CBD/kg bw/day for liver toxicity in humans [73] were taken as reference. Assuming that consumers follow dosage guidance found via the Internet (see also Section 7), it is evident that the typical starting dose (0.5–30 mg/day) is already in the range of or even above the provisional ADI of 10 mg/day. Standard dosages (10–115 mg/day) exceed the provisional ADI by up to 10-fold and include doses associated with evidence for drug–drug interactions in humans (70 mg/day). Macro dosage recommendations (50–1500 mg/day) not only exceed the provisional ADI by more than two orders of magnitude but are also close to and even far above the LOAEL for liver toxicity in humans. Figure 2 serves to illustrate how easily the ADI and even the LOAEL for liver toxicity may be reached by consumers via consumption of CBD oils currently available on the market:

- **ADI of 10 mg/day for a 70 kg person (corresponding to 0.14 mg/kg bw/day) [24]**.

According to the UK FSA, 4–5 drops of an oil-based supplement containing 5% CBD of 98% purity is approximately equivalent to the provisional ADI of 10 mg CBD/day for an average 70 kg adult [24]. Assuming a 20% CBD oil of 98% purity, a single drop is sufficient to reach the ADI. Therefore, the ADI is easily reached or even exceeded with just a few drops of CBD oil, depending on the CBD content of the product used. It should be considered that this even applies to products with a low CBD content of 5%. Some of the foods currently on the market therefore contain significantly more than 10 or 12 mg CBD per serving.

- **LOAEL for liver toxicity, 300 mg/day (corresponding to 4.3 mg/kg bw/day for a 70 kg person).**

To reach the LOAEL for liver toxicity of 300 mg/day, which is also the therapeutic starting dose, approximately 30 drops of a product containing 20% CBD at 98% purity would be required. However, assuming the worst case, i.e., an oil-based food supplement with a CBD content of up to 40% and a purity of 98%, only 15 drops would be sufficient to lead to exposure in the range of the LOAEL. Additionally, consumption of only seven drops of a product containing 20% CBD at 98% purity results in an exposure approximately equivalent to the LOAEL of 1 mg/kg bw/day (70 mg for a 70 kg adult) reported for inhibitory drug–drug interactions [24]. Therefore, exposure in the range of the LOAEL for liver toxicity or for drug-drug interactions can easily be reached by consumers of oil-based food supplements.

- **Therapeutic dose range of 600–1000 mg/day (corresponding to approx. 9–14 mg/kg bw/day for a 70 kg person)**

Consuming oil-based food supplements containing high amounts of up to 40% CBD with a purity of 98%, it is possible to reach the therapeutic dose range of 600–1000 mg CBD/day. It can be assumed that a daily intake of 30–50 drops of such a product is easily achievable by high consumers.

Considering that CBD exposure is likely to exceed the provisional ADI even at recommended starting doses, the SKLM concluded that consumption of CBD-containing food supplements may pose a health risk to consumers.

## 9. Benefit Characterization

Numerous CBD products are advertised as food supplements with various health claims. In Europe, such health claims on foods require scientific evidence, and no claims for CBD have been registered or approved to date. The SKLM evaluated the available evidence, particularly from human studies, to determine whether CBD as a pure substance has potential health benefits in a dose range below the therapeutic starting dose for Epidyolex^®^ of 300 mg/person/day (4.3–5 mg/kg bw/day).

The present evaluation shows that there is currently no convincing evidence for beneficial effects of CBD on recovery and performance, sleep quality and insomnia, the immune system, cardiovascular/heart health, positive mood and good cognitive function, and for its neuroprotective effects in healthy volunteers at doses below 300 mg/day (<4.3–5 mg/kg bw/day). Some of the health claims, e.g., “positive mood”, “relaxation”, or “stress resistance”, are considered only as vague terms for health claims and are therefore not sufficiently characterized for a scientific evaluation. The literature review did not reveal a single study on CBD that examined the endpoint “relaxation”. Thus, there is currently a lack of evidence suggesting that CBD (at any dose) helps or maintains relaxation beyond placebo effects. There are almost no data on menstrual discomfort, especially on associated abdominal pain, and on endometriosis. Due to the limited data available, a specific conclusion on CBD and arthritis pain, joint pain, or arthrosis pain is currently not possible. Of the various beneficial effects studied with CBD, anxiety relief has been suggested as the most replicable result at doses of 300–400 mg/day (approx. 4.3–5.7 mg/kg bw/day). However, there is currently a lack of evidence for effects on anxiety in the 100–150 mg dose range (approx. 1.4–2.1 mg/kg bw/day) and a lack of informative studies below 100 mg/day (approx. 1.4 mg/kg bw/day). Therefore, anxiety-related claims are currently not sufficiently demonstrated for the low dose range of CBD expected in food supplements.

In summary, there is currently no convincing scientific evidence that any of the claimed beneficial effects of CBD evaluated here can be induced/expected in healthy humans at doses below 300 mg/day (<4.3 mg/kg bw/day), which are in line with standard dosage recommendations. It should be noted that there are only a few and often preliminary human studies in healthy individuals available in this dose range. Under pathological conditions and in the higher therapeutic dose range above 300 mg CBD/day (>4.3 mg/kg bw/day), there is some evidence that specific beneficial effects might be induced, e.g., on the cardiovascular system, sleep, or anxiety. However, due to possible concomitant adverse effects, this dose range can only be considered for medical applications (i.e., pharmaceutical products) and not for food/food supplements.

## 10. Assessment—Risk–Benefit Integration

In human studies, adverse effects on the liver were reported at 4.3–5 mg/kg bw/day (considering a body weight of 70 or 60 kg, respectively), while the NOAEL is unknown. As the ACNFP/COT noted, there is evidence in humans that oral intake levels above 70 mg CBD/day may lead to adverse interactions with certain drugs in some individuals [66]. The committees also noted that a dose of 70 mg CBD/day was the lowest dose investigated in human studies and that therefore drug interactions cannot be excluded at doses below 70 mg/day [66]. NOAELs in animal studies were reported at 20–72 mg/kg bw/day [66,278]. Based on human and/or animal data, derived ADI values or daily doses that should not be exceeded, respectively, were in good agreement at 10–12 mg CBD/day [24,25,278]. The SKLM concurs with these ADI values that should not be exceeded. In addition, according to the ACNFP/COT, pregnant and breastfeeding women and patients on prescription medication should not consume products containing CBD [66]. The SKLM agrees with this recommendation.

No scientifically justified beneficial effects were observed in healthy humans at doses below 300 mg/day. Therefore, based on the available data, no beneficial effects (as claimed in the various health claims) are to be expected at acceptable intake levels at which no risk is to be expected. Accordingly, the health claims with which the CBD products are marketed (e.g., on the internet) are currently not scientifically substantiated and justified. The consumption of commercially available CBD products could even lead to health risks for the consumer. Despite several uncertainties in estimated exposures, the calculation of different intake scenarios indicates that exposures in the range of ADI levels or even in the range of the LOAEL for liver toxicity or for drug–drug interactions would easily be reached by consumers of oil-based food supplements with an average CBD content. With oil-based food supplements containing high amounts of CBD, it is even possible for consumers to reach doses in the therapeutic range of 600–1000 mg CBD/day.

As the initial step of the risk–benefit analysis clearly shows that the risks outweigh the benefits when considering exposure scenarios relevant for food supplements, a further refined risk–benefit assessment was not carried out.

## 11. Conclusions

The SKLM concludes that there is a lack of evidence of a benefit for any of the health claims revisited here at doses below 300 mg CBD/day, and that there may be a risk of liver toxicity and possible drug–drug interactions in this dose range. In view of the evidence of possible drug–drug interactions, there is concern about the potential concomitant use of medicinal drugs. Irrespective of the absence of beneficial effects, it is recommended to avoid an exceedance of the provisional ADI of 10 mg/day for a 70 kg person set by the FSA [24]. It can be assumed that a healthy consumer will not be harmed by this amount of CBD in its pure form (≥98% purity). However, numerous CBD-containing food supplements currently on the market provide more than 10 mg CBD per serving. The health claims used for these products are not scientifically substantiated, and moreover, they also lack the required novel food authorization. More importantly, depending on the CBD content and dosage recommendations, exposure to CBD from such products may easily exceed ADI levels and even the LOAEL for liver toxicity. Consequently, the SKLM considers that consumption of CBD-containing food supplements may pose a risk to human health, and therefore risk communication is needed to raise awareness of the issue.

## 12. Research Needs

To perform a comprehensive risk–benefit assessment, as suggested by EFSA [28], more reliable data on benefits and risks are needed. Future studies should consider existing data and follow standardized and internationally accepted criteria (depending on the in vitro, animal, or human study, e.g., OECD or ARRIVE (Animal Research: Reporting of In Vivo Experiments)). Raw data and study protocols need to be provided, at least upon request.

With regard to potential risks, several uncertainties that should be clarified have already been defined by EFSA [2] and ACNFP/COT [66], e.g., concerning the bioavailability of the pure form of CBD, the effects of chronic life-time use, the existence of vulnerable subgroups, and potential CBD–drug interactions [2,66]. Furthermore, there are data gaps in particular with regard to reproductive toxicity and immune toxicity/immunosuppression.

## Figures and Tables

**Table 1:** Brief overview of adverse health effects of CBD.

Organ System/Endpoint		Adverse Effects		Dose Range/Conditions		References
Liver		Increased liver weights Liver cell hypertrophy Elevated liver enzymes (ALT, AST, ALP, GGT), increased bilirubin Potential drug-induced liver injury (DILI)		Animal LOAEL (lowest): 10 mg/kg bw/day (dogs) Human LOAEL: 4.3 mg/kg bw/day		[2,43,44,45,46,47,48]
						
Gastrointestinal		Diarrhea (dose-dependent, up to 57% occurrence) Upset stomach Flatulence Abdominal cramps		400–1500 mg/day (5–21.4 mg/kg bw/day) More frequently in fasted state		[46,49,50,51,52,53,54,55,56]
						
Neurological/Psychiatric		Headache Somnolence Dizziness Attention disturbance Insomnia Nightmares Mental sedation Lethargy Ataxia Abnormal motor coordination Irritability Aggression, anger		Most effects seen at 1500 mg/day (~20 mg/kg bw/day), some already at 5 mg/kg bw/day. Some effects at 400 mg single dose (~6 mg/kg bw); e.g., increase in mental sedation		[2,18,48,54,57]
						
Endocrine		Inhibition of prolactin, growth hormone, luteinizing hormone, estradiol, progesterone, testosterone, vasopression secretion Decreased T4 and T3 levels Increased TSH Thyroid follicular cell hypertrophy Changes in gonadotropin levels Effects on sex hormone levels Adrenal gland toxicity		Hypothalamic-pituitary-gonadal axis: Effects from 30 mg/kg bw/day in animals Thyroid effects from 80–100 mg/kg bw/day in animals		[2,18,43,44,45,58,59,60,61]
						
Reproduction/Fertility		Decreased gonadal weight Reduced testes size (8–25%) Inhibited spermatogenesis Decreased sperm quality Sperm morphological abnormalities Reduced fertility rates Reduced fetal weights Increased prenatal losses		Effects from 15–30 mg/kg bw/day in animals		[18,44,45,59,60,61,62]
						
Drug Metabolism		Inhibition of multiple CYP enzymes Inhibition of UGT1A9 and UGT2B7 Drug–drug interactions		Effects at clinically relevant concentrations Drug–drug interactions observed at 1 mg/kg bw/day		[2,17,63,64,65,66]
						
Genotoxicity		Negative Ames tests, in vivo micronucleus and comet assays Clastogenic or aneugenic effects at the site of first contact cannot be ruled out		Ames test: ≤5000 µg/plate negative In vitro micronucleus assay: ≤11 µg/mL negative In vivo micronucleus assay: ≤1000 mg/kg bw/day negative In vivo comet assay: 500 mg/kg bw/day negative		[2,18,67,68]

ALT: alanine aminotransferase; AST: aspartate transaminase; ALP: alkaline phosphatase; GGT: gamma-glutamyl transferase; CYP: cytochrome P450; LOAEL: lowest observed adverse effect level; T3: triiodothyronine; T4: thyroxine; TSH: thyroid-stimulating hormone; UGT: UDP-glucuronosyltransferase.

**Table 2:** The impact of CBD on physical performance.

Study Design		CBD Dose *		Source		Route		Duration		Effects		Comment/Limitations		Reference
Healthy men, 18–45 years old (*n* = 10); randomized, double-blind, placebo-controlled, crossover design		300 mg/day (4.3 mg/kg bw/day)		Formulation of synthetic CBD		Oral		Single administration		Increase of VO2 max Ratings of pleasure		The changes were small, making it difficult to reliably evaluate the effect of CBD.		[89]
														
Healthy men and women, 18–42 years old (*n* = 48); randomized, double-blind, placebo-controlled		50 mg/day (0.7 mg/kg bw/day)		Hemp-derived CBD		Oral		8 weeks		Placebo group experienced a decline in mean peak power and relative peak power compared to the CBD group. CBD did not affect body composition, aerobic fitness, muscular strength, physical activity, cognitive health, psychological wellbeing, and resting CRP concentrations.		Physical activity and exercise training were not monitored nor evaluated during the intervention period.		[90]
														
Trained men, 22–24 years old (*n* = 7–8 per group); not blinded		16.67 mg/day (0.24 mg/kg bw/day)		CBD oil, no information on purity		Oral		Single administration		CBD group reported significant differences in VAS score at post-EIMD.		Small sample size, self-reported scores.		[91]
														
Untrained men, 21.85 ± 2.73 years old (*n* = 13); double-blinded, placebo controlled, crossover design		150 mg/day (2.14 mg/kg bw/day)		CBD oil		Oral		3 days		Dose of 150 mg CBD oil had no effect on non-invasive markers of muscle damage in the upper extremity 24 h and 48 h after the intervention.		Only non-invasive markers have been determined.		[92]
														
Female athletes 21.2 ± 1.8 years old (*n* = 24); double-blind, placebo-controlled, crossover design		224–408 mg per application (5 mg/kg bw/day)		Not clear		Oral		3 times in 10 h		No differences for muscle damage markers. Subjective fatigue as measured by the VAFS was not significantly different (*p* > 0.05) between the CBD and placebo. CBD supplementation was unable to reduce fatigue and restore performance.		Published conference abstract, not peer-reviewed article.		[93]
														
Trained men and women, 24 ± 3 years old (*n* = 21); randomized, double-blind, placebo-controlled, study in a six-arm crossover design		60 mg/person (0.86 mg/kg bw/day)		Plant derived		Oral		Single application		No significant effects in biomarkers or performance parameters. Small and significant effects of a single supplementation of CBD on CK and Myo concentrations after 72 h.				[94]
														
Highly trained male weightlifters, 25 ± 3 years old (*n* = 12); double-blind, placebo-controlled, study in two-arm crossover design		60 mg/person (0.86 mg/kg bw/day)		Plant derived		Oral		Single application		Significant effects of a single supplementation with CBD on CK and Myo concentrations after 24 h. Reduced strength 24 h after CBD consumption.		Pilot study, only 12 participants. Experiment is also one arm of the follow-up study by Isenmann et al., 2021.		[95]
														
Healthy men, 18–65 years old (*n* = 40; 20 per group); randomized, double-blind, placebo-controlled, repeated-dose pilot study		70 mg/person (1 mg/kg bw/day) and 100 mg CBG per day		Water soluble liquid. CBD oil nano-particularized and combined with other ingredients to maintain the suspension.		Oral		3.5 days		Minor effects on decreased self-reported average soreness/discomfort 72 h post-DOMS. No meaningful effects on objective measures of recovery.		CBD was given in combination with CBG. Effects only on markers which are self-reported.		[96]
														
Healthy men and women, 18–40 years old (*n* = 17, m = 15, f = 2); randomized, double-blind, placebo-controlled in a three-arm cross-over design		60 mg/person (0.86 mg/kg bw/day)		CBD oil solubilized CBD		Oral		6 days		CBD oil reduced skeletal muscle damage in highly trained athletes. Significant decrease in platelets lymphocyte ratios in advanced athletes after placebo treatment (*p* < 0.05). CBD oil application showed a slight inhibitory effect. CBD products do not affect performance and inflammatory parameters.		Study was performed with athletes of different performance levels. Wash out phase was only 4 weeks.		[97]

* Calculated for a person with 70 kg bw. CBD: cannabidiol; CBG: cannabigerol; CK: creatine kinase; CRP: C-reactive protein; DOMS: delayed-onset of muscle soreness; EIMD: exercise-induced muscle damage; VAS: visual analogue scale; VAFS: Visual Analogue Fatigue Scale (VAFS); VO2 max: maximum rate of oxygen consumption; Myo: myoglobin.

**Table 4:** The impact of CBD on the immune system.

Study Design		CBD Dose *		Source		Route		Duration		Effects		Comment/Limitations		Reference
Healthy adults (*n* = 10); pilot, randomized, double-blind, parallel arm study		30 mg/day (0.43 mg/kg bw/day)		Commercially available water- and lipid-soluble CBD powders.		Oral		Single application		TNF-α was decreased in LPS-stimulated PBMCs collected 90 min after CBD exposure relative to cells collected at baseline.		Pilot study		[138]
Healthy men and women, 18–42 years old (*n* = 48); randomized, double-blind, placebo-controlled		50 mg/day (0.7 mg/kg bw/day)		Hemp-derived CBD; no information about purity.		Oral		8 weeks		CBD did not affect resting CRP concentrations.		Study was conducted to examine training effects (i.e., inflammation not main investigated parameter).		[90]

* Calculated for a person with 70 kg bw. CBD: cannabidiol; CRP: C-reactive protein; LPS: lipopolysaccharide; PBMC: peripheral blood mononuclear cells; TNF-α: tumor necrosis factor α.

**Table 6:** The impact of CBD on anxiety.

Study Design		CBD Dose *		Source		Route		Duration		Effects		Comment/Limitations		Reference
Healthy men and women (*n* = 60); randomized, double-blind, placebo-controlled		100, 300, 900 mg/day (1.4, 4.3, 12.9 mg/kg bw/day)		99.6% purity in corn oil filled in gelatin capsules		Oral		Single administration		The subjective anxiety measures based on a test of public speaking in a real situation followed by determination of anxiety and sedation factors of the VAS for mood were reduced with 300 mg CBD, but not with 100 and 900 mg.		Acute administration CBD dose not adapted to subject’s weight Subjective measurements of anxiety Due to the small sample size limiting the statistical power, the finding of U-shaped dose response curve needs to be interpreted with caution.		[194]
Healthy men (*n* = 57); randomized, double-blind, placebo-controlled		150, 300, 600 mg/day (2.1, 4.3, 8.6 mg/kg bw/day)		99.9% purity in corn oil filled in gelatin capsules		Oral		Single administration		The subjective anxiety measures based on a test of public speaking in a real situation followed by determination of ratings on the VAS for mood were reduced with 300 mg CBD, but not with 150 and 600 mg.		Acute administration CBD dose not adapted to subject’s weight Subjective measurements of anxiety Due to the small sample size limiting the statistical power, the finding of U-shaped dose response curve needs to be interpreted with caution.		[195]
Healthy men (*n* = 45); randomized, double-blind, placebo-controlled		150 mg/day (2.1 mg/kg bw/day)		99.6% purity either as powder or dissolved in corn oil, filled in gelatin capsules		Oral		Single administration		The responses to stimuli during a facial emotion recognition task were altered with neither of the CBD formulations.		Acute administration CBD dose not adapted to subject’s weight Subjective measurements of anxiety Small sample size with limited statistical power		[196]
Healthy men and women (*n* = 32); randomized, double-blind, placebo-controlled		150, 300, 600 mg/day (2.1, 4.3, 8.6 mg/kg bw/day)		CBD isolate (< 0.3% THC), no purity stated, in MCT oil with 2% peppermint oil		Oral		Single administration		No effect on self-reported test anxiety or general anxiety		Acute administration CBD dose not adapted to subject’s weight Subjective measurements of anxiety Small sample size with limited statistical power		[207]
Male and female outpatients with moderate to severe anxiety (*n* = 14); open-label stage of a two-stage, phase 2 clinical trial (unblinded study)		About 30 mg/day (0.43 mg/kg bw/day)		Full-spectrum high CBD extract in MCT oil		Oral		30 days		Anxiety determined using self-report scales was significantly reduced at week 4 relative to baseline. Clinical response was obtained as early as week 1 in most patients.		Various other cannabinoids in the source CBD dose not adapted to subject’s weight Subjective measurements of anxiety Small sample size with limited statistical power Transferability from patients to general consumers questionable Bias from open label design towards treatment expectations		[208]
Men and women with elevated trait worry (*n* = 63); randomized, double-blind, placebo-controlled		50 mg CBD/day (0.7 mg/kg bw/day) with 0.03 mg CBDV, 300 mg CBD/day (4.3 mg/kg bw/day)		CBD isolates in MCT oil in soft gel capsules		Oral		2 weeks		No effects of acute CBD dosing Repeated administration of 300 mg but not 50 mg reduced self-reported anxiety symptoms		CBD dose not adapted to subject’s weight Subjective measurements of anxiety Small sample size with limited statistical power		[209]
Healthy men and women (*n* = 48); randomized, double-blind, placebo-controlled		32 mg/day (0.46 mg/kg bw/day)		CBD (purity not specified) in ethanol vehicle		Inhalation		Single administration		CBD may be an adjunct to extinction-based therapies for anxiety disorders.		Inhalation study not directly transferrable to foods CBD dose not adapted to subject’s weight Small sample size with limited statistical power Anxiety not a direct endpoint of study design		[210]
Audit of patients (*n* = 400) seeking CBD prescriptions		40–300 mg/day (0.6–4.3 mg/kg bw/day)		Pharmaceutical CBD oil (100 mg/mL in 25 mL bottles)		Oral		3 weeks		Patients with mental-health-related symptoms experienced improvements in self-reported anxiety (*p* = 0.02). Patients with neurological symptoms, pain symptoms, and cancer symptoms experienced no statistically significant differences.		Dosing and compliance unclear Subjective measurements of anxiety No control group Bias from open label design towards treatment expectations		[211]
Retrospective case series (*n* = 72)		25 mg/day (0.34 mg/kg bw/day)		CBD (purity not specified) in capsule form		Oral		3 months		Anxiety scores decreased within the first month in 57 patients (79.2%) and remained decreased during the study duration.		Dosing and compliance unclear Subjective measurements of anxiety No control group Bias from open label design towards treatment expectations		[212]
Female patient, 10 years old (*n* = 1); case report		12–25 mg/day (0.17–0.36 mg/kg bw/day)		Sublingual spray, capsules		Oral		6 months		CBD may be effective to reduce anxiety-provoked sleep disorder after a traumatic experience.		Subjective measurements of anxiety Bias from open-label design towards treatment expectations		[213]

* Calculated for a person with 70 kg bw. CBD: cannabidiol; CBDV: cannabidivarin; MCT: medium-chain triglyceride; VAS: visual analogue scale.

**Table 7:** The impact of CBD on stress symptoms.

Study Design		CBD Dose *		Source		Route		Duration		Effects		Comment/Limitations		Reference
Female patients with irritable bowel syndrome, 22–50 years old (*n* = 32); randomized, double-blinded, placebo-controlled, crossover design		50 mg/day (0.7 mg/kg bw/day)		CBD-containing chewing gum		Oral		8-week		There was no statistically significant difference in pain scores between CBD and placebo at a group level. Subgroup and individual analyses showed a highly variable picture.		Markers for the direct response of the immune system to CBD were not investigated in this study.		[232]
8 groups of 5 male volunteers (*n* = 40)		Single dose of 15 to 60 mg (0.2–0.9 mg/kg bw/day) with single dose of Δ^9^-THC		Substance solved in orange juice		Oral		Single dose		Ingestion of Δ9-THC together with CBD induced weaker stress and anxiety.				[224]
Patients with clinical high risk for psychosis (*n* = 32) and healthy controls (*n* = 26)		600 mg/day (8.6 mg/kg bw/day)		Capsules		Oral		1 week		Change in cortisol level was associated with experimental stress exposure in healthy volunteers.				[203]
Healthcare workers during the COVID-19 pandemic (*n* = 120)		300 mg/day (150 mg twice per day) (4.3 mg/kg bw/day)		Dissolved in medium-chain triglyceride		Oral		4 weeks		Improvement of burnout syndrome		4 participants treated with CBD experienced elevated liver enzymes and 1 participant severe pharmacodermia		[72]
Healthcare workers (*n* = 13) during the COVID-19 pandemic (27 July–28 November 2020)		330 mg CBD/day, (165 mg twice per day) (4.7 mg/kg bw/day)		Dissolved in medium-chain triglyceride		Oral		4 weeks		Improvement of burnout syndrome symptoms and other mental health outcomes				[201]
11 patients with a diagnosis of PTSD; retrospective case series		Open-label, flexible dosing regimen: 25 to 100 mg CBD/day together with psychiatric medications and psychotherapy (0.3–1.4 mg/kg bw/day)		In oil or capsules		Oral		8 weeks		Decrease in PTSD symptom severity		Other treatments in parallel		[230]

* Calculated for a person with 70 kg bw. CBD: cannabidiol; PTSD: post-traumatic stress disorder.

**Table 8:** The impact of CBD on sleep.

Study Design		CBD Dose *		Source		Route		Duration		Effects		Comment/Limitations		Reference
Human														
Healthy men and women, 29 ± 8.5 years old (*n* = 26); randomized, double-blind, placebo-controlled, crossover design		300 mg/day (4.3 mg/kg bw/day)		Pure compound, 99.9% purity		Oral		Single administration		CBD did not induce any significant effect regarding cognitive and subjective measurements related to sleep (*p* > 0.05).				[246]
Healthy men and women, 18–55 years old (*n* = 296; *n* = 63 to 56 per group); randomized, double-blind, placebo-controlled		20 mg CBN, (c) 20 mg CBN + 10 mg CBD (0.14 mg/kg bw/day), (d) 20 mg CBN + 20 mg CBD (0.3 mg/kg bw/day), or (e) 20 mg CBN + 100 mg CBD (1.4 mg/kg bw/day)		Gummies pure compounds		Oral		7 days		Individuals receiving 20 mg CBN demonstrated reduced nighttime awakenings and overall sleep disturbance relative to placebo, with no impact on daytime fatigue. Concomitant CBD administration did not positively augment CBN treatment effects.		CBD was not investigated without CBN.		[247]
Healthy men and women; 25.8 ± 6.1 years old (*n* = 28; *n* = 14 per group); double-blind, placebo-controlled intervention		50 mg/day (0.7 mg/kg bw/day)		Purified hemp-derived CBD		Oral		8 weeks		No significant differences between groups with respect to mental health measures, sleep quantity, or circulating immunophenotype. CBD significantly improved sleep quality, as measured by a sleep questionnaire.		Sleep quality was self-reported by questionnaire.		[248]
Healthy men and women, 45 ± 12 years old (*n* = 1793; *n* = 300 per group); 6 arm, randomized, double-blinded, controlled study		6 groups combining 15 mg CBD/day (0.2 mg/kg bw/day) with other cannabinoids and/or melatonin: I. 15 mg CBD + 15 mg CBN + 5 mg melatonin II. 15 mg CBD + 15 mg CBN III. 15 mg CBD IV. 5 mg melatonin V. 15 mg CBD full spectrum + 15 mg CBN VI. 15 mg CBD + 15 mg CBN + 5 mg CBC		Not clear. Mixed quality (pure, from hemp extract/full spectrum)		Oral		5 weeks		Clinically relevant improvement in the sleep quality of most participants (56% to 75%) across all administered formulations. No significant differences between 15 mg CBD isolate and all other formulations. Chronic use of CBD at a low dose is safe and could improve sleep quality, though these effects do not exceed those of 5 mg melatonin.		No placebo control Variable dose Sleep quality self-reported online Effect of CBD was comparable to an effect of 5 mg melatonin.		[249]
Healthy men and women, 18–45 years (*n* = 30; *n* = 15 per group); randomized, placebo-controlled, parallel design		150 mg/day (2.1 mg/kg bw/day)		Pure CBD		Oral		2 weeks		Insomnia severity, subjective sleep onset latency, sleep efficiency, and wake after sleep onset did not differ between treatments throughout the trial (all *p* > 0.05).		Good experimental design Sleep parameters were measured, not based on self-report.		[250]

* Calculated for a person with 70 kg bw. CBC: cannabichrome; CBD: cannabidiol; CBN: cannabinol.

**Table 10:** Range of CBD dose recommendations found on the Internet (see also Appendix A, Table A1, and [274]).

Dosage Type	CBD Dose (mg/day)	Possible Usage Pattern for CBD-Oils *
***Micro dosage***	0.5–30	5% CBD-oil: < 1 drop–12 drops 10% CBD-oil: < 1 drop–6 drops 20% CBD-oil: << 1 drop–3 drops 40% CBD-oil: <<< 1 drop–1.5 drops
***Standard dosage***	10–115	5% CBD-oil: 4–46 drops 10% CBD-oil: 2–23 drops 20% CBD-oil: 1–11.5 drops 40% CBD-oil: < 1 drop–6 drops
***Macro dosage***	50–1500	5% CBD-oil: 20–600 drops 10% CBD-oil: 10–300 drops 20% CBD-oil: 5–150 drops 40% CBD-oil: 2.5–75 drops

* Assuming the following CBD content in one drop of oil (50 µL): 5% CBD-oil—2.5 mg; 10% CBD-oil—5 mg; 20% CBD-oil—10 mg; 40% CBD-oil – 20 mg. The different shades of orange indicate increasingly high CBD dose, starting from Micro dosage (light orange-lowest CBD dose) to macro dosage (dark orange-highest CBD dose).

## Data Availability

The original contributions presented in this study are included in the article and Appendix A. Further inquiries can be directed to the corresponding author.

## Appendix A

**Table A1:** Examples of online dosing recommendations for CBD oils (websites accessed 20 November 2024). Dosage recommendations on the internet often refer to low-, medium-, and high-dose ranges used for different health conditions. CBD dosage regimen is usually based on the following publication: Leinow L. and Birnbaum J. (2019) Heilen mit CBD: Das wissenschaftlich fundierte Handbuch zur medizinischen Anwendung von Cannabidiol. München: Riva Verlag [275]. A slow dose adjustment or gradual dose increase is usually recommended. Dosages are often given together with health claims or supposedly appropriate indications for a range of diseases or conditions. It is important to note that these health- and disease-related claims are not approved for food and that most of the effects are not evidence-based.

Website	Low (Micro) Dosage ^1^ [mg/day]	Medium (Standard) Dosage ^1^ [mg/day]	High (Macro) Dosage ^1^ [mg/day]	Recommended Dosage Range [mg/day]
https://flavorfix.com/cbd/cbd-dosage-chart-calculator/ ^2^ (accessed on 15 November 2024)				180–909
https://www.verywellmind.com/cbd-dosages-how-much-cbd-should-you-take-5078580 (accessed on 15 November 2024)				Anxiety: 300–600 Bowel disease: 10 Cancer-related pain: 50–600 Parkinson’s disease: 75–300 Poor sleep: 25 Psychosis: 600
https://www.kurkliniken.de/blog/cbd-oel-richtig-dosieren-darauf-kommt-es-bei-der-anwendung-an.html (accessed on 15 November 2024)				20–1500
https://www.forbes.com/health/cbd/cbd-dosage/ (accessed on 15 November 2024)				Anxiety: 300–600 Select forms of epilepsy: starting at 2.5 mg/kg bw/twice daily Central neuropathic and cancer-related pain: maximum of 30 Opioid addiction: 400 or 800 Arthritis: maximum of 30 (or 250 if applied topically)
https://www.praktischarzt.de/ratgeber/cbd-oel/dosierung/ (accessed on 15 November 2024)	0.5–20	20–100	400	
https://naturalcbd.at/dosierung-von-cbd/ ^3^ (accessed on 15 November 2024)	1–32	15–115	75–1500	
https://www.cbd-vital.de/magazin/cbd-allgemein/cbd-dosierung (accessed on 15 November 2024)	1–20	10–100	50–800	
https://www.calconic.com/calculator-widgets/cbd-dosage-calculator ^4^ (accessed on 15 November 2024)	10–20	15–30	25–50	
https://cbd360.de/cbd-oel-dosierung/ (accessed on 15 November 2024)	2–20	20–80	80–800	
https://cbdsfinest.de/magazin/cbd-oel-dosierung-einnahme/ (accessed on 15 November 2024)	0.5–20	10–100	50–800	
https://www.benetui.com/de/magazin/wie-viel-mg-cbd-pro-tag-soll-ich-nehmen ^4^ (accessed on 15 November 2024)	14 ^5^	49 ^5^	91 ^5^	Pain: 2.5–20 Anxiety/stress: 5–30 Sleep disorder: 40–160 Epilepsy: 200–300
https://cannatrust.eu/wiki/cbd-dosierung/ ^6^ (accessed on 15 November 2024)	10–25	30–75	60–150	
https://cbd-deal24.de/was-ist-cbd/cbd-oel-dosierung/ (accessed on 15 November 2024)	0.5–25	10–100	50–800	
https://www.cbd-guru.co.uk/cbd-beginners-guide/?srsltid=AfmBOoqnpU082CprHEzOyFGyStiEb_HeWZ3VQa9vxEfcZ8Ro_Lybi1u8 (accessed on 15 November 2024)	0.5–20	10–100	50–800	
https://www.naturalwayscbd.com/blog/cbd-dosage-chart/ ^7^ (accessed on 15 November 2024)	8–24	24–72	40–120	

^1^ The CBD oil doses recommended online are typically divided into three dosage ranges: low (i.e., micro), standard (i.e., medium), or macro (i.e., high) dosage based on body weight and/or the severity of symptoms. ^2^ Recommended doses given considering a body weight ranging from 36 kg to 100 kg. ^3^ Recommended doses given considering a body weight ranging from 45 kg to 105 kg. ^4^ The amount of CBD suggested is based on the severity of the condition (mild, medium, severe). ^5^ Refers to a person of 70 kg body weight. ^6^ Recommended doses given considering a body weight ranging from 45 kg to 110 kg. ^7^ Recommended doses given considering a body weight ranging from 36 kg to 109 kg.
